# Overexpression of miR-125a in Myelodysplastic Syndrome CD34^+^ Cells Modulates NF-κB Activation and Enhances Erythroid Differentiation Arrest

**DOI:** 10.1371/journal.pone.0093404

**Published:** 2014-04-01

**Authors:** Irene Gañán-Gómez, Yue Wei, Hui Yang, Sherry Pierce, Carlos Bueso-Ramos, George Calin, María del Carmen Boyano-Adánez, Guillermo García-Manero

**Affiliations:** 1 Department of Systems Biology, University of Alcalá, Alcalá de Henares, Madrid, Spain; 2 Department of Leukemia, The University of Texas MD Anderson Cancer Center, Houston, Texas, United States of America; 3 Department of Hematopathology, The University of Texas MD Anderson Cancer Center, Houston, Texas, United States of America; 4 Department of Experimental Therapeutics, Division of Cancer Medicine, The University of Texas MD Anderson Cancer Center, Houston, Texas, United States of America; Cincinnati Children's Hospital Medical Center, United States of America

## Abstract

Myelodysplastic syndromes (MDS) are characterized by impaired proliferation and differentiation of hematopoietic stem cells. The participation of toll-like receptor (TLR)-mediated signaling in MDS is well documented. Increased TLR signaling leads to the constitutive activation of NF-κB, which mediates inflammation, cell proliferation and apoptosis. In addition, the TLR pathway induces the expression of miRNAs which participate in the fine-tuning of the inflammatory response. miRNAs also regulate other biological processes, including hematopoiesis. miR-125a and miR-125b are known modulators of hematopoiesis and are abnormally expressed in several hematologic malignancies. However, little is known about their role in MDS. NF-κB-activating ability has been described for both miRNAs. We studied the role of miR-125a/miR-125b in MDS and their relationship with TLR signaling and hematopoietic differentiation. Our results indicate that miR-125a is significantly overexpressed in MDS patients and correlates negatively with patient survival. Expression of miR-99b, which is clustered with miR-125a, is also directly correlated with prognosis of MDS. Both miR-125a and miR-99b activated NF-κB *in vitro*; however, we observed a negative correlation between miR-99b expression and the levels of TLR2, TLR7 and two downstream genes, suggesting that NF-κB activation by the miRNA cluster occurs in the absence of TLR signaling. We also show that TLR7 is negatively correlated with patient survival in MDS. In addition, our data suggest that miR-125a may act as an NF-κB inhibitor upon TLR stimulation. These results indicate that miR-125a is involved in the fine-tuning of NF-κB activity and that its effects may depend on the status of the TLR pathway. Furthermore, we observed that miR-125a inhibits erythroid differentiation in leukemia and MDS cell lines. Therefore, this miRNA could serve as a prognostic marker and a potential therapeutic target in MDS.

## Introduction

Myelodysplastic syndromes (MDS) are a heterogenous group of clonal hematologic malignancies associated with hematopoietic stem cell dysfunctions which lead to impaired cell proliferation and differentiation [1]. The presence of increased apoptosis in bone marrow (BM), mainly due to augmented death receptor signalling [2], and the frequent transformation to acute myeloid leukemia (AML) [1] are other distinctive features of this group of diseases. Increasing incidence and significant mortality rates, especially in higher-risk cases [1]–[3], make it necessary to establish prognostic factors and more selective therapies for MDS, but the high clinical and cytogenetic heterogeneity and the not well-understood molecular mechanisms of these malignancies complicate this task [2]–[4]. Furthermore, progress in MDS research has been hampered up to now by the lack of fully-characterized cell lines from patients and the difficulties in the development of mouse models [2].

Toll-like receptors (TLRs) are the most important type of mammalian pattern recognition receptors participating in the innate immunity response [5], [6]. Upon stimulation, TLRs signal through adaptor molecules like the myeloid differentiation primary response gene 88 (MyD88), ultimately inducing the transcription of interferons and proinflammatory cytokines through the activation of transcription factors like NF-κB. Importantly, TLRs located in monocytes/macrophages may also upregulate the expression of genes involved in metabolism, tissue repair and differentiation [5], [7], [8]. The presence of TLRs in BM stem and progenitor cells (CD34^+^) and their involvement in the modulation of myeloid differentiation has been extensively reported [7], [9], [10] and there is compelling evidence that TLR signaling is important in the pathogenesis of MDS (reviewed in [8]). Moreover, TLR1, TLR2, TLR4 and TLR9 have been found to be overexpressed in BM of MDS patients [11]–[14]. This overexpression appears to be correlated with the prognosis of the disease, and with the levels of the cytokine TNF-α, thought to be responsible for intramedullary cell death in MDS [11]–[14]. Furthermore, we recently reported MyD88 overexpression [15] and identified a gain-of-function mutation of TLR2 [14], [16] in MDS CD34^+^ cells. Both alterations are involved in a positive feedback loop which results in the constitutive expression of NF-κB and ultimately leads to the production of inflammatory cytokines, the increase of the cell proliferation rate and the blockade of differentiation of BM progenitors [14]–[16].

In addition to the expression of pro-inflammatory factors, TLR signaling also induces the expression of microRNAs (miRNAs) which participate in the fine-tuning of the inflammatory response [5], [8], [17]. miRNAs are conserved, non-coding short RNAs that participate in post-transcriptional regulation by binding to mRNAs, generally to their 3′-untranslated region (UTR), and blocking protein expression [18]. The majority of human miRNA genes are isolated from each other within the genome, but others can be clustered together and transcribed as a polycistronic primary miRNA transcript [18], [19]. Because miRNAs within a genomic cluster have similar or the same expression patterns, they are often functionally related to each other [18]. For instance, one target mRNA can bear binding sites for many different miRNAs, which is indicative of their collaborative mechanism of action [20]. In consequence, miRNAs have cell-type and tissue-specific expression patterns, as well as distinct expression signatures in multiple pathologies, like cancer [18], [19], [21]. Because miRNAs participate in the control of hematopoiesis, their deregulation also appears to be involved in the pathogenesis of several hematopoietic diseases [22]. Multiple miRNAs have been reported to be abnormally expressed in hematologic cancers, especially in lymphoma, myeloma and chronic lymphocytic leukemia [21], [23]–[28]. Moreover, specific miRNA expression profiles have been proposed as diagnostic and prognostic markers in various hematologic malignancies [26], [29], [30], including MDS [31]–[33]. Therefore, abnormal TLR signaling in MDS could be influenced by the expression of a specific miRNA signature, which in turn could participate in the pathogenesis of the disease through modulation of hematopoiesis and inflammatory response.

miR-125a and miR-125b are two of the most-studied miRNAs participating in the regulation of hematopoiesis and cell differentiation [34]–[37] and there is novel evidence of their involvement in lymphoid and myeloid diseases [37]–[42]. They are located in homologous miRNA clusters encoded in chromosomes 19 and 11, respectively. Recurrent chromosomal translocations affecting the locus of miR-125b and resulting in a strong overexpression of this miRNA have been reported in MDS and AML [38], [40]. However, little is still known about the participation of miR-125a in the pathogenesis of MDS. Interestingly, NF-κB-activating ability has been described for both miR-125a and miR-125b [43], and the promoter of miR-125b is known to contain an NF-κB binding-site [44] and to be upregulated by NF-κB activation [45]. This suggests the participation of miR-125b in a positive feedback loop within the NF-κB pathway. This fact, along with the recent finding that secreted miRNAs can bind and directly activate TLRs [46], make miR-125a and miR-125b candidates to play a role in the regulation of the TLR/MyD88/NF-κB axis and therefore participate in the molecular mechanisms involved in MDS. For these reasons, the aims of the present work were to analyze the role of miR-125a and miR-125b in MDS and to study their relationship with TLR signaling pathways and with cell differentiation, and to explore their utility as prognostic markers and therapeutic targets for this group of diseases.

## Materials and Methods

### Ethics statement

Human MDS bone marrow specimens were obtained from patients referred to the Department of Leukemia at MD Anderson Cancer Center following protocol LAB01-473, which was approved by MD Anderson's Institutional Review Board (IRB) 5. Written informed consent was obtained from donors. All the cell lines used are from human origin and have been published before.

### Primary samples

MDS bone marrow cells were collected from 48 MDS patients. Diagnosis was confirmed by a dedicated hematopathologist as soon as a sample was obtained. Patient characteristics are summarized in Table S1 (Supporting information). Human bone marrow cells from 6 healthy individuals were obtained from AllCells (Emeryville, CA). Isolation of CD34^+^ cells was performed using MicroBead Kit (Miltenyi, Bergisch Gladbach, Germany), following manufacturer's instructions.

### Cell lines

The human chronic myelogenous leukemia cell lines Meg-01 [47] and K562 [48] were obtained from ATCC (Manassas, VA) and cultured in RPMI medium with L-glutamine, supplemented with 10% (v/v) FBS and 1% (v/v) penicillin/streptomycin (HyClone, Thermo Fisher Scientific, Waltham, MA). The human myelodisplastic syndrome cell line MDS-L [49] was kindly provided by Dr. Starczynowski (Cincinnati Children's Hospital, Cincinnati, OH, USA) and cultured in RPMI medium with L-glutamine, supplemented with 20% (v/v) FBS, 1% (v/v) penicillin/streptomycin and 10 ng/mL human IL-3 (HyClone, Thermo Fisher Scientific, Waltham, MA). The AML cell line KG1 [50] was also obtained from ATCC and cultured in IMDM medium supplemented with 20% (v/v) FBS and 1% (v/v) penicillin/streptomycin (HyClone, Thermo Fisher Scientific, Waltham, MA). All cell cultures were maintained at 37°C in an atmosphere with 5% CO_2_. The acute myeloid leukemia cell lines HL-60 [51], THP-1 [52] and OCI/AML3 [53] were used exclusively for nucleic acid extraction to assess their miRNA levels. HL-60 and THP-1 cells were obtained from the ATCC (Manassas, VA, USA) and grown in RPMI medium, supplemented with 10% (v/v) heat-inactivated FBS and 1% (v/v) penicillin/streptomycin; OCI/AML3 were provided by Dr. Bueso-Ramos (Department of Hemopathology, MD Anderson Cancer Center, Houston, TX, USA) and cultured in RPMI medium, supplemented with 20% (v/v) heat-inactivated FBS and 1% (v/v) penicillin/streptomycin (all from HyClone, Thermo Fisher Scientific, Waltham, MA, USA).

### Treatments

Cytarabine (Ara-C; Sigma-Aldrich Co., St. Louis, MO) was stored at −20°C as a 1 mM stock solution in PBS. Final concentration in cell cultures was 1 µM. In combination treatments, Ara-C was added to growth medium 6 hours after the treatment with miRNA inhibitors.

The MyD88 inhibitory peptide (Pepinh-MYD) and its control peptide (Pepinh-Control; both from Invivogen, San Diego, CA, USA) were dissolved in sterile PBS to prepare 1 mM stock solutions, which were stored at −20°C. Final concentration in cultured cells was 5 µM. In combination treatments, peptides were added to growth medium 2 hours after the treatment with miRNA inhibitors.

Lipopolysaccharide (LPS) and PAM(3)CysSK(4) (PAM3) (both from Invivogen, San Diego, CA, USA) are specific ligands (agonists) of TLR4/TLR2 [365] and TLR2 [79], respectively. LPS and PAM3 were dissolved in sterile PBS for a working concentration of 1 mg/mL and stored at −20°C. Final concentrations in cell cultures were 1 µg/mL for LPS and 0.1 µg/mL for PAM3.

### RNA isolation and reverse transcription (RT)

After collection, cells were washed with PBS and lysed with Trizol (Invitrogen, Carlsbad, CA) according to the manufacturer's specifications for RNA extraction. Final, purified RNA was dissolved in sterile distilled water. cDNA was synthesized using High Capacity cDNA Reverse Transcription Kit (Applied Biosystems, Carlsbad, CA), according to the manufacturer's protocol and using 400 ng of RNA per reaction. To obtain miRNA-specific cDNA, random primers were replaced with the corresponding TaqMan miRNA-Expression Assays (Applied Biosystems, Life Technologies, Carlsbad, CA).

### Quantitative real-time PCR (qPCR)

qPCR was performed using TaqMan Expression Assays for TLR7, erythropoietin receptor (EPO-R), glycophorin A (GYPA), CD71 or transferrin receptor (TFRC), PU.1 (SPI1), integrin γ-M (ITGAM) and GAPDH genes and miRNA-Expression Assays for miR-125a, miR-125b, miR-99b, let-7e and snU6 (Applied Biosystems, Carlsbad, CA), according to the manufacturer's protocol. cDNA for protein-coding gene assays was diluted 1∶5 or 1∶10 prior use. qPCR reactions were performed with TaqMan Universal PCR Mastermix (Applied Biosystems, Carlsbad, CA) in a 7500 Real-Time PCR System (Applied Biosystems, Carlsbad, CA). Each condition was run in triplicates to minimize variability. Expression levels of protein-coding genes and miRNAs were normalized to those of GAPDH and snU6, respectively.

### Lipid-based transfection

Meg-01 cells were transfected with the pGL4.32[luc2P/NF-κB-RE/Hygro] Vector (Promega, Madison, WI), containing an NF-κB response element (NF-κB-RE) that drives transcription of the luciferase reporter gene *luc2P*, along with the pRL-TK vector (Promega, Madison, WI), containing the transfection control reporter gene *Rluc*. The negative control (“mock” control) #1 mirVana miRNA mimic, the hsa-miR-99b Pre-miR miRNA Precursor or the hsa-miR-125a-5p mirVana miRNA mimic (all from Applied Biosystems, Carlsbad, CA) were co-transfected with the luciferase vectors at the concentration of 50 nM. Every transfection reaction was performed in the presence of Lipofectamine 2000 and OptiMEM medium (Invitrogen, Carlsbad, CA). All luciferase assay experiments (n = 3) were plated in duplicates and each sample was measured twice to minimize the high variability inherent to this technique.

### Electroporation

Cell Line Nucleofector Solution R (Lonza, Basel, Switzerland) was used to nucleofect KG1 cells with pGL4.32[luc2P/NF-κB-RE/Hygro] and pRL-TK vectors (Promega, Madison, WI) using program V-001 of Amaxa Nucleofector and the corresponding reagent kit (Lonza, Basel, Switzerland). Two hours after nucleofection, KG1 cells were treated with anti-sense oligonucleotides (ASO; see below) and, after two more hours, with LPS and PAM3.

### Luciferase reporter gene assays

After 48 hours from transfection (Meg-01 cells) or 24 hours from nucleofection (KG1), cells were collected, washed with PBS and lysed with Passive Lysis Buffer (Promega, Madison, WI) according to the manufacturer's protocol for Active Lysis. Luciferase activity of the lysates was then measured using the Dual-Luciferase Reporter Assay kit (Promega, Madison, WI) in a Monolight 3010 luminometer (Pharmingen, Beckton-Dickinson Biosciences, San Diego, CA).

### miRNA inhibition with anti-sense oligonucleotides

Anti-sense oligonucleotides (ASO) are efficient biological tools for the inhibition of miRNA activity, *in vivo* and *in vitro* [54]. An ASO for miR-125a was designed following the guidelines by Horwich and Zamore [55], along with a specific control ASO (sequence details provided in Table S2), and synthesized by Dharmacon (Thermo Scientific, Waltham, MA). For miRNA inhibition dose-response assays, KG1, K562 and MDS-L cells were cultured at a cell density of 5×10^4^ cells/mL, treated with ASO concentrations between 100 nM and 1 µM and incubated for 48 hours prior analysis of miRNA levels and cytotoxicity of ASOs. For colony formation assays, K562 were treated with either Ara-C 6 hours after treatment with 1 µM ASO and MDS-L cells were treated with 5 µM of Pepinh-MYD and its respective control peptide 2 hours after treatment with ASO.

### Determination of cell number and viability

After incubation with ASO, K562 and MDS-L cells were counted in a phase-contrast microscope with 1∶1 trypan blue 0.4% (Sigma-Aldrich, St. Louis, MO). The number of cells was counted twice for every independent experiment and the mean value was used in the statistical analysis of all the experiments.

### Methylcellulose colony formation assays

After 48 hours of incubation with ASO, 250 cells of each condition were resuspended in 0.5 mL of methylcellulose MethoCult GF H4434 (Stem Cell Technologies, Vancouver, BC, Canada), diluted with the respective culture medium, and plated in duplicates in and for a 12-well dish. A variable number of wells were filled with PBS to preserve humidity in the plate and prevent methylcellulose from dehydration. Cells were allowed to grow at 37°C and 5% CO_2_ for at least 4 days for K562 cells and 7 days for MDS-L cells, after which colonies were counted in a phase-contrast microscope and subsequently collected for RNA extraction.

### Benzidine staining

Erythroid differentiation of K562 cells can be determined by heme group staining with benzidine [56]. A volume 1∶1 of working solutions containing 0.2% benzidine (Sigma-Aldrich, St. Louis, MO), 0.5% acetic acid and 0.6% H_2_O_2_ was added to each well and heme-positive colonies (blue) were counted after 1–3 minutes in a phase-contrast microscope.

### Statistical analysis

Overall survival (OS) was defined from date of initial diagnosis to date of death. To investigate associations between gene expression and OS, we considered splitting expression level at the 25^th^, 50^th^ and 75^th^ percentiles, generating three possible binary variables. Differences in miRNA expression levels between patients and controls were analyzed using the non-parametric Mann-Whitney test. Linear correlations between miRNA levels or between TLR pathway mRNA levels were determined using the Pearson or Spearman correlation tests, where appropriated. In other cases, two cohorts of patients were established based on relative miRNA levels, as “higher” or “lower” than the mean of all patients. Mann-Whitney test was used to analyze the differences between the two groups. Outliers were removed using Grubb's or ROUT methods. The rest of the statistical analyses were carried out by individual Student t tests, one- or two-way ANOVA, as required. Data are presented as mean ± SEM of at least three independent experiments. Statistical significance: *P<0.05; **P<0.01; ***P<0.001; ****P<0.0001.

## Results

### miR-125a and miR-125b are overexpressed in MDS CD34^+^ cells

In order to find out if miR-125a and/or miR-125b play a relevant role in the pathogenesis of MDS, their relative expression in CD34^+^ cells from BM of MDS patients was analyzed by qPCR and compared to their expression in BM CD34^+^ cells from healthy donors.

**Figure 1:**
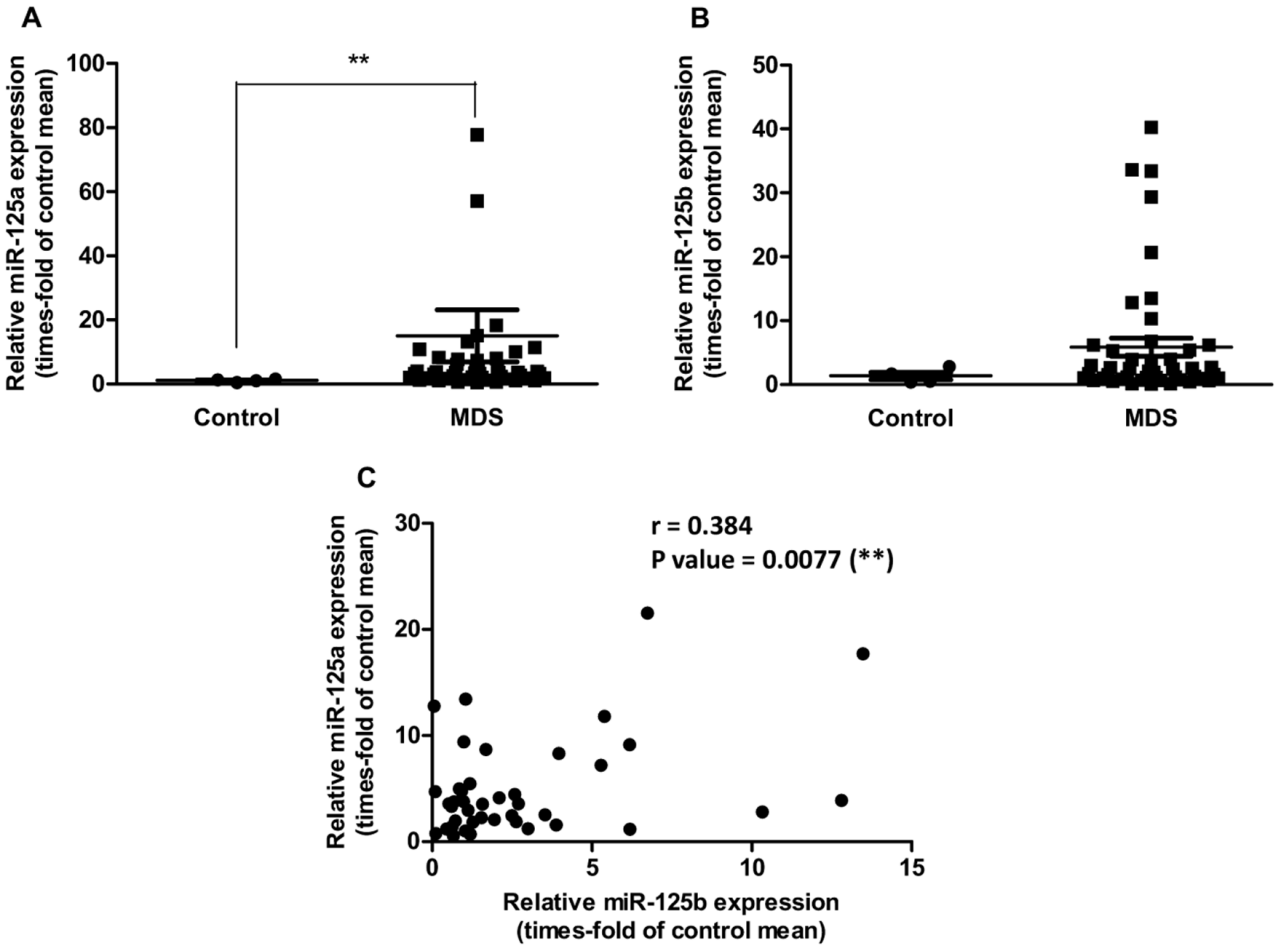
Expression of miR-125a and miR-125b in BM CD34^+^ cells. (**A–B**) Relative expression of miR-125a and miR-125b in CD34^+^ cells from MDS patients (N = 48/47, respectively) and healthy donors. In (**A**), one data point (Y = 386.31) is outside the axis limits. Statistical significance versus healthy donors: **P<0.01. (**C**) Correlation between relative expression of miR-125a and miR-125b in MDS CD34^+^ cells. Six data points are outside the axis limits.

As shown in Figure 1A, miR-125a was significantly overexpressed in MDS patients when compared with healthy controls, with relative expression levels higher than 2-fold in 71% of the patients. miR-125b also had a trend towards overexpression in MDS patients, with a relative expression higher than 2-fold in 49% of them; however differences with healthy controls were not statistically significant (Figure 1B). Levels of both miRNAs were correlated in a direct fashion (Figure 1C), which suggests that they might be partially subjected to the same regulatory mechanisms. Of note, miR-125a expression levels were twice as high as miR-125b levels in MDS patients and healthy controls (data not shown), which might be indicative of a more important regulatory function for miR-125a.

Previous studies of potential miRNA signatures in MDS had reported that miR-125a [57], [58] and miR-125b [38], [57], [58] are upregulated in MDS with 5q deletion. We did not detect any significant difference in the expression of miR-125a or miR-125b between MDS with a 5q alteration and other MDS (data not shown), although this might be due to the fact that only 5 of our patients presented with 5q deletions. Likewise, miR-125b overexpression and its tumorigenic activity have been reported exclusively in MDS patients with translocations in chromosome 11 (q23 and q24) [38], [40]. No patients in our cohort presented with translocations in the aforementioned locus, suggesting that miR-125b overexpression is not necessarily associated with chromosomal aberrations.

### miR-125a, but not miR-125b, is significantly correlated with survival in MDS

To determine if the overexpression of these two miRNAs is clinically relevant in MDS, we next studied the correlation between patient survival and levels (“high” or “low”) of miR-125a and miR-125b.

**Figure 2:**
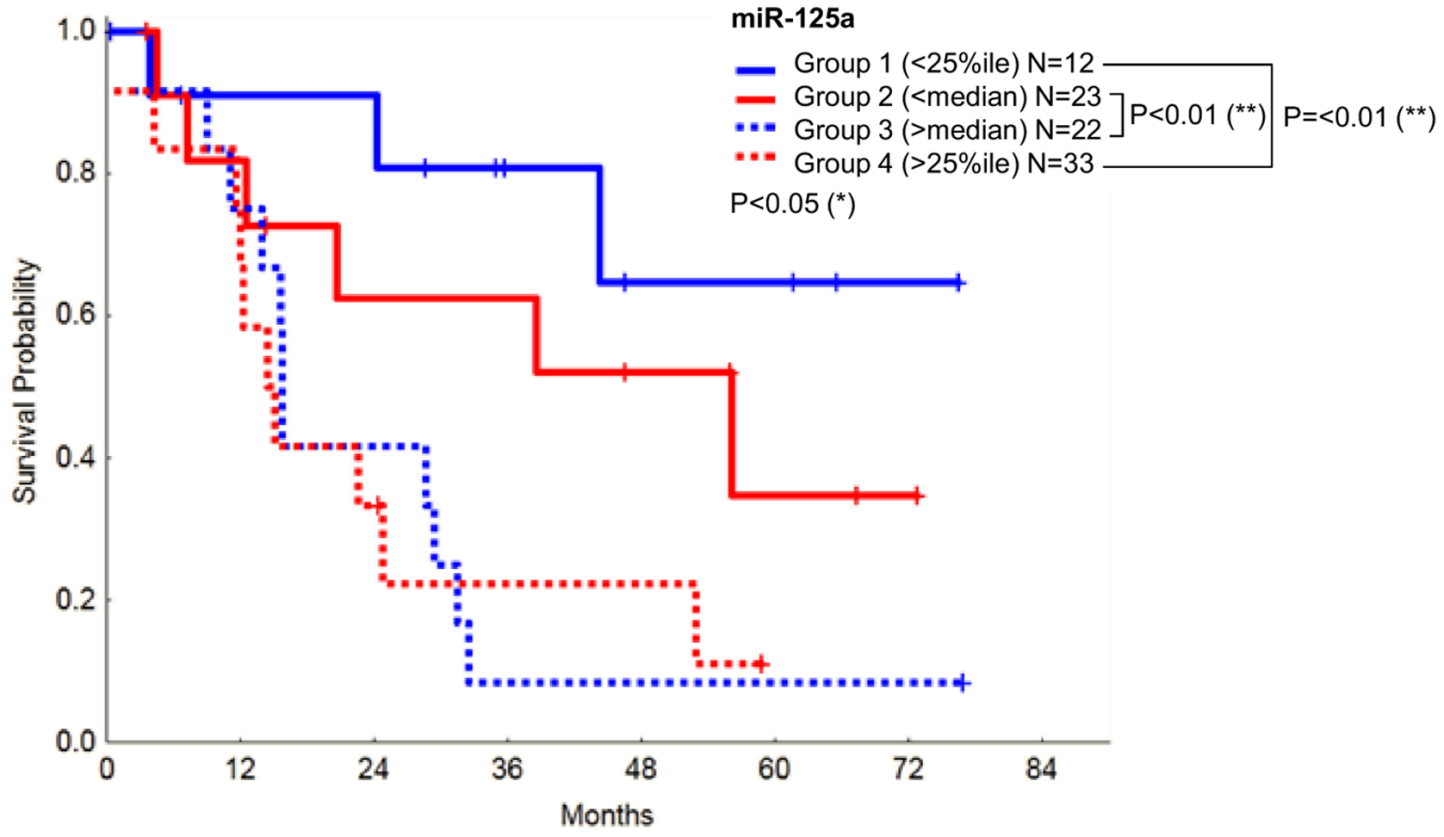
Correlation between the relative expression of miR-125a and overall survival in MDS. Levels of miR-125a are inversely correlated with patient survival.

Expression of miR-125a was significantly correlated with prognosis (Figure 2), with patients with higher miRNA levels showing a poorer prognosis. These results confirm the clinical relevance of miR-125a in MDS. In contrast, miR-125b expression did not appear to have a significant impact on the overall survival of MDS patients (data not shown).

### The miR-99b/let-7e/miR-125a cluster may be involved in the pathogenesis of MDS

In light of the clinical data, we focused on the study of miR-125a. This miRNA is encoded in a conserved DNA cluster comprising the sequence of two more miRNAs: miR-99b and let-7e (Figure S1). In order to determine if the other members of the cluster are involved in MDS, we also analyzed their relative expression in patient CD34^+^ cells.

**Figure 3:**
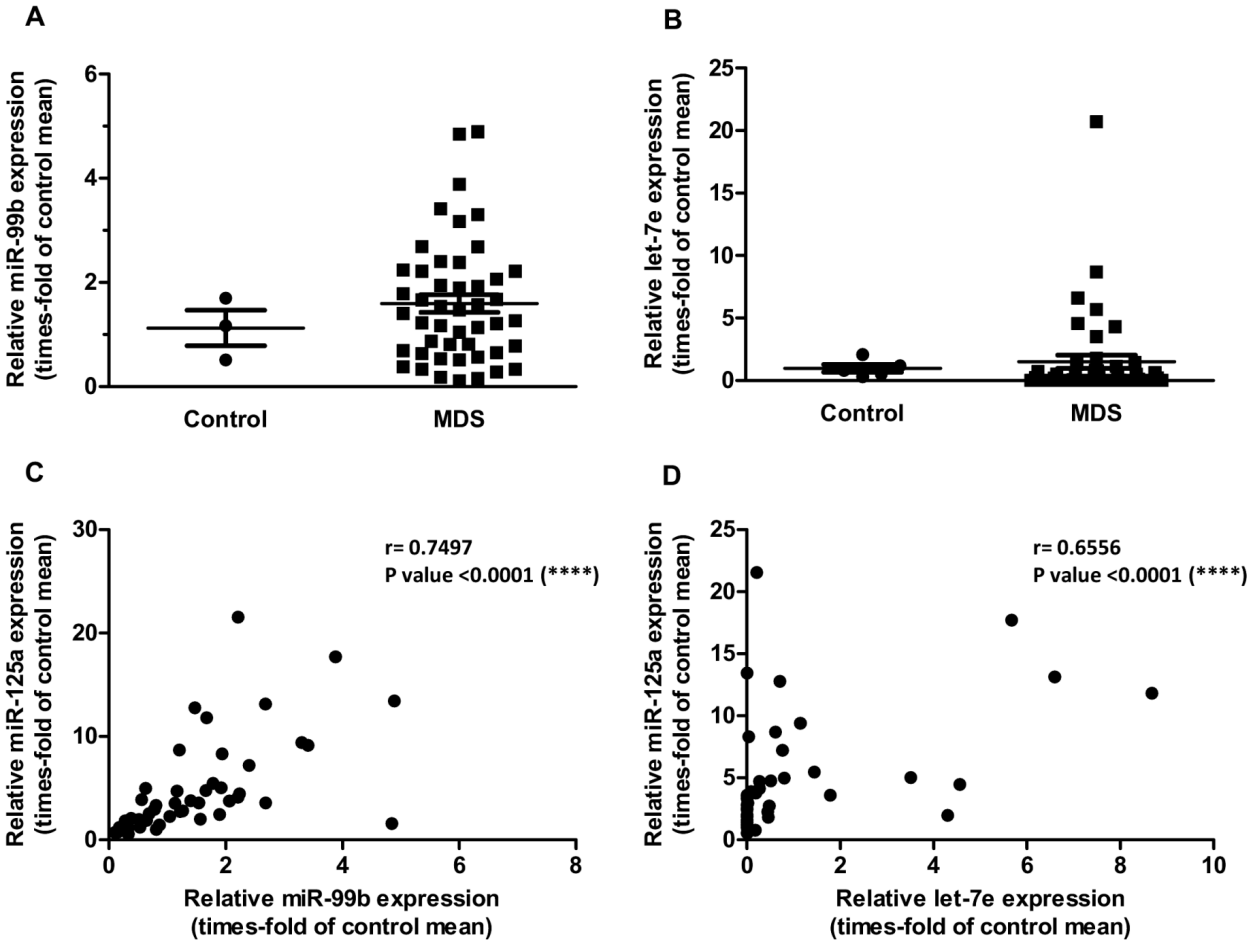
Expression of miR-99b and let-7e in BM CD34^+^ cells. (**A–B**) Relative expression of miR-99b and let-7e in CD34^+^ cells from MDS patients (N = 48/45, respectively) and healthy donors. One outlier removed by Grubb's method (α = 0.05) in each case. (**C–D**) Correlation between the relative expression of miR-125a and miR-99b or let-7e, respectively, in MDS CD34^+^ cells. In (C) and (D), three data points are outside the axis limits.

Although there were no statistically significant differences with the control group (Figure 3A), increased miR-99b (over 2-fold of controls) was present in 30% of patients and strongly correlated with miR-125a levels (Figure 3C), indicating co-expression of both cluster members. let-7e was the least abundant miRNA of the cluster, with low expression in controls and barely detectable levels in most patients (data not shown). It is unclear if this fact is a result of an inefficient detection of the miRNA by our qPCR experiments or a consequence of a defective miRNA processing or a faster decay. Again, there were no significant differences between patients and healthy donors (Figure 3B) and this time only 18% of patients expressed let-7e over 2-fold of controls. However, let-7e levels directly correlated with the expression of both miR-125a (Figure 3D) and miR-99b (r = 0.394, P = 0.0075; data not shown).

**Figure 4:**
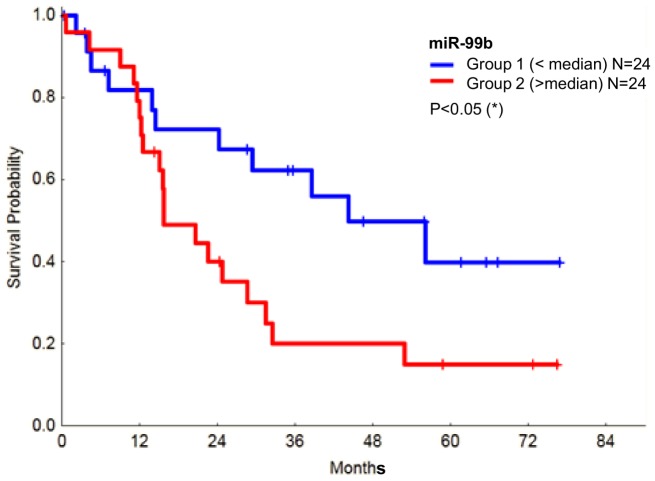
Correlation between the relative expression of miR-99b and overall survival in MDS. Levels of miR-99b are inversely correlated with patient survival.

Interestingly, survival studies revealed that expression of miR-99b (Figure 4), but not that of let7-e (data not shown), is significantly correlated with a poor prognosis. However, it needs to be taken into account that this could be a consequence of the strong correlation existing between the relative expression of miR-125a and miR-99b.

Altogether, data indicate that the expression of the members of the miR-99b/let-7e/miR-125a cluster is regulated by a common mechanism and that both miR-99b and miR-125a are clinically relevant in MDS. However, the considerably higher expression levels of miR-125a suggest that, during its biogenesis, it undergoes alternative mechanisms of regulation which allow it to accumulate independently of the rest of the cluster, and that this miRNA could be the most important cluster component in the pathogenesis of MDS.

### miR-125a and miR-99b are positive regulators of NF-κB activity *in vitro*

Since both miR-125a and miR-99b appeared to be clinically relevant in MDS, we sought to investigate their possible connection with the TLR/NF-κB pathway. For this purpose, we co-transfected Meg-01 cells with a reporter vector containing NF-κB response elements and with synthetic analogs of both miRNAs, and determined their activity on NF-κB activation through the luciferase reporter gene assay.

**Figure 5:**
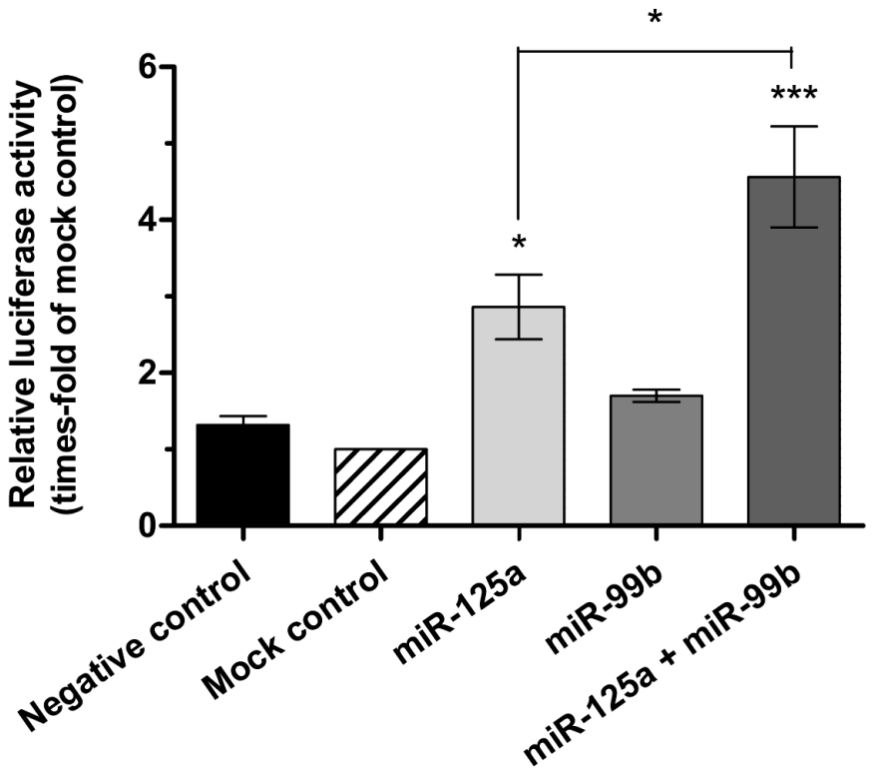
Effect of ectopic expression of miR-125a and/or miR-99b on NF-κB activity. NF-κB activation was determined after 48 hours from transfection of Meg-01 cells with miRNA mimics and luciferase vectors. Results are relative to cells transfected with mock RNA and expressed as mean ± SEM of n = 3 independent experiments. Statistical significance: *P<0.05; ***P<0.001.

As shown in Figure 5, ectopic expression of miR-99b was only able to induce a slight increase on NF-κB activity, while miR-125a significantly augmented it. Because we showed before that it is likely that both miRNAs are co-expressed in MDS cells, the combination of both of them was also studied. Interestingly, ectopic expression of miR-125a and miR-99b induced a more powerful activation of NF-κB when co-transfected together, indicating that they can cooperate in a synergistic manner. Nevertheless, miR-125a appears to drive most of the NF-κB-activating activity.

These results agree with previous observations that miR-125a activates NF-κB through the repression of the TNF-induced NF-κB inhibitor TNFAIP3 [43]. Furthermore, an integrated search of the target-prediction bioinformatic algorithms DIANA microT [59], miRanda [60], [61], Pic Tar [62] and TargetScanS [63] in the database miRGen [64] predicted two other NF-κB inhibitors as potential miR-125a targets: TNIP2, which is a TNFAIP3-binding protein, and NF-κB inhibitor epsilon, also known as IκBε. Similarly, a miRGen search revealed NF-κB repressing factor (NFKRF) as a potential target of miR-99b. Thus, it is possible that miR-125a and miR-99b activate NF-κB through the specific repression of inhibitors of this pathway.

### Expression of miR-99b and miR-125b is inversely correlated with the levels of the TLR2/NF-κB pathway members

The hyperactivation of the TLR2/MyD88/NF-κB pathway in MDS CD34^+^ cells has been recently reported by our group [14]–[16]. Provided that miR-125a and miR-125b are potential NF-κB regulators [43] and in light of the results presented above, we next studied the associations between miR-125b or the miR-99b/let-7e/miR-125a cluster and the expression of TLR2, MyD88 and the histone demethylase JMJD3, which we had identified as a positive regulator of inflammation downstream of NF-κB, that could participate in a positive feedback loop resulting in the hyperactivation of this factor [14]. Expression levels of TLR2, MyD88 and JMJD3 in this subset of patients had already been determined by our group and published elsewhere [14]–[16].

**Figure 6:**
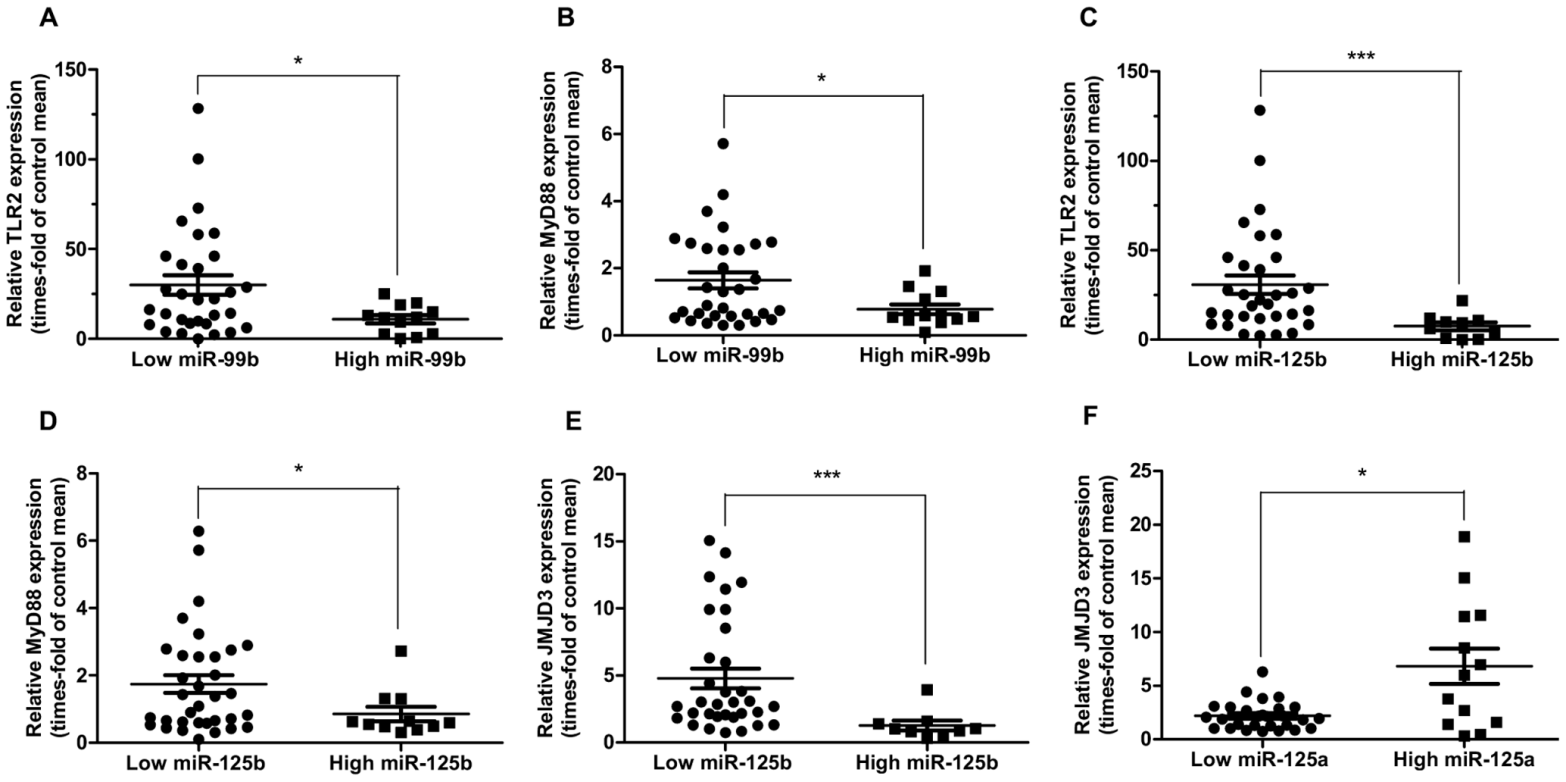
Relationship between the miR-99b/let-7e/miR-125a cluster members and genes from the TLR2-NF-κB pathway in MDS. “Low” and “high” expression cohorts were established based on comparison with the mean relative expression value. (**A–B**) Correlation between the relative expression of miR-99b and TLR2 or MyD88, respectively, in MDS CD34^+^ cells. In (B), one outlier removed by ROUT method. (**C–E**) Correlation between the relative expression of miR-125b and TLR2, MyD88 or JMJD3, respectively in MDS CD34^+^ cells. In (E), five outliers removed by ROUT method. (**F**) Correlation between relative expression of miR-125a and JMJD3. Seven outliers removed by ROUT method. Statistical significance: *P<0.05; ***P<0.001.

As shown in Figure 6, miR-99b and miR-125b levels are negatively correlated with TLR2 and MYD88 expression, and miR-125b also negatively correlates with JMJD3 levels. These results suggest that miR-99b and miR-125b are only overexpressed in those MDS patients in which the TLR/NF-κB pathway is not hyperactivated. Because our experimental data and the literature indicate that both miR-99b and miR-125b [43] are potential NF-κB activators, the negative association with members of the TLR signaling axis could indicate that these two pathways of NF-κB activation are mutually exclusive. However, no correlations were found between miR-125a and most of the genes studied (data not shown). These differences between miR-99b and miR-125a support our previous hypothesis that the latter undergoes different mechanisms of regulation than the rest of the members of the cluster and miR-125b, and may implicate that mature miR-125a can be upregulated simultaneously with TLR pathway members in MDS.

It is worth noting that a significant positive correlation was found between miR-125a levels and the expression of JMJD3 (Figure 6F). This histone demethylase is regulated by the TLR/NF-κB pathway and, in turn, positively regulates the expression of many NF-κB target genes [14]. Nevertheless, because those are not the only genes subjected to epigenetic regulation by JMJD3, the expression of this enzyme is not exclusively modulated by innate immunity pathways, as indicated by its correlation with TLR2 (r = 0.365, P = 0.016) and MYD88 levels (r = 0.437; P = 0.002). Thus, JMJD3 can be upregulated also in those patients with low or normal TLR/MYD88 levels. For this reason, it is possible that the expression of miR-125a and JMJD3 in MDS is associated independently of TLR signaling. It is tempting to hypothesize that JMJD3 could positively regulate miR-125a transcription. Nevertheless, the positive correlation between the two genes is not strong enough to assume that they depend on each other and, furthermore, no significant association was found with miR-99b (Figure S2A). Therefore, it is unlikely that there is a causal relationship between miR-125a and JMJD3 and the positive correlation observed is probably a consequence of the elevated expression of both genes in most MDS patients.

### TLR7 expression is deregulated and correlates with a better prognosis in MDS

Because it was recently postulated that miRNAs can bind and activate endogenous TLRs such as TLR7 and TLR8 [46], and we had previously detected overexpression of TLR7 in a small cohort of MDS CD34^+^ patients [16], we determined to investigate if the regulation of this TLR is also altered in MDS and if it is connected with miR-125a/miR-99b expression.

**Figure 7:**
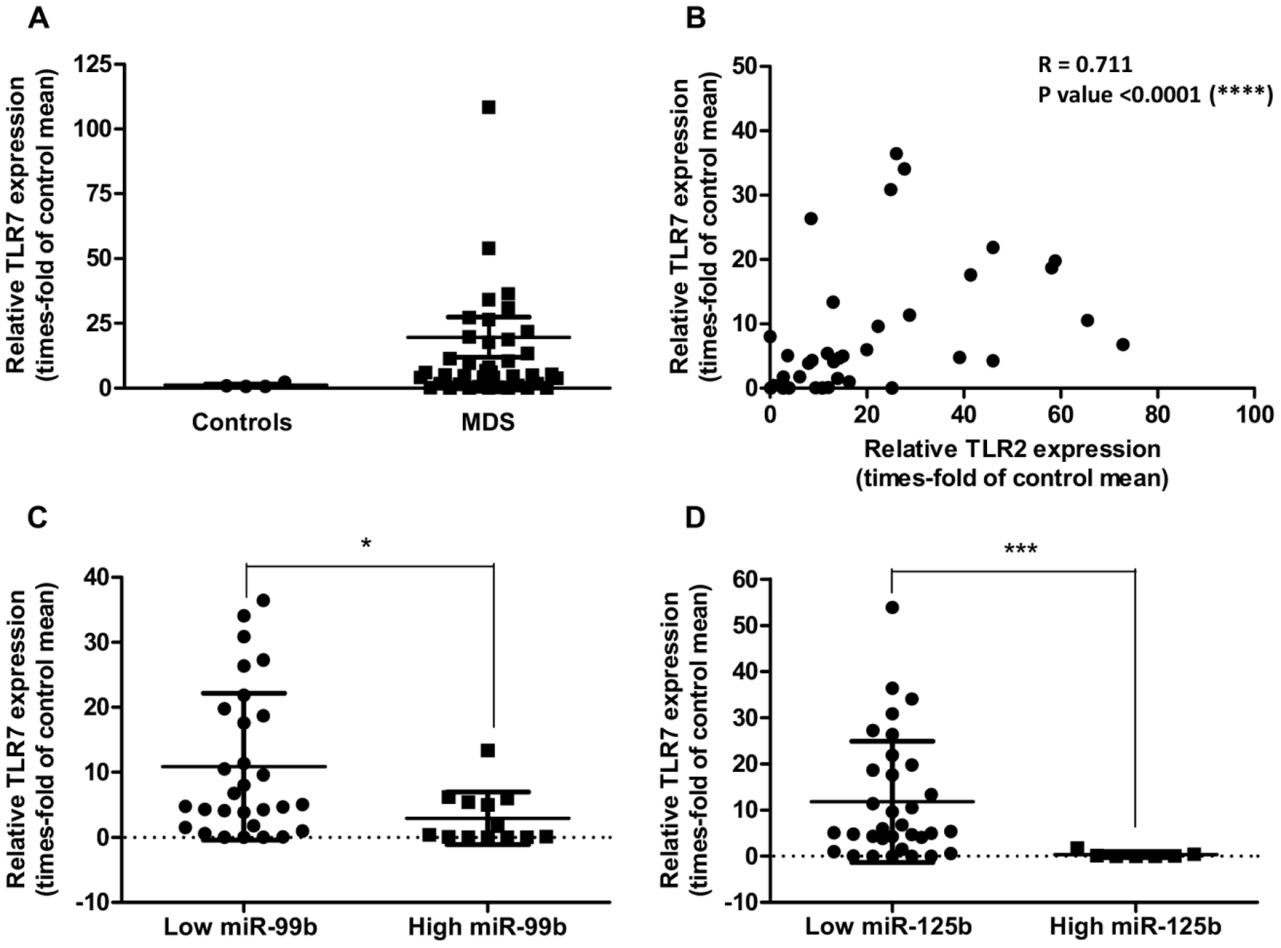
Expression of TLR7 in MDS. (**A**) Relative expression of TLR7 in BM CD34^+^ cells. mRNA levels of TLR7 in CD34^+^ cells from MDS patients (N = 42) and healthy donors were measured by qPCR. (**B–E**) Correlation between the relative expression of TLR7 and TLR2, miR-99b, miR-125b or miR-125a, respectively, in MDS CD34^+^ cells. “Low” and “high” expression cohorts were established based on comparison with the mean relative expression value. In (B), two data points are outside the axis limits; in (C), three outliers removed by ROUT method; in (D), four outliers removed by ROUT method; in (E), seven outliers removed by ROUT method. Statistical significance: *P<0.05; ***P<0.001.

Relative expression levels of TLR7 in MDS CD34^+^ cells were higher than 2-fold of healthy controls in 64.44% of patients; however, differences between groups were not statistically significant (P = 0.0932) (Figure 7A). Remarkably, we found a strong direct correlation (P<0.0001) between TLR7 and TLR2 expression levels in these patients (Figure 7B), which suggests that TLR2 and TLR7 may be simultaneously upregulated in MDS. Of note, the association of TLR7 expression with other members of the pathway like MYD88 or JMJD3 (r = 0.361, P = 0.017; and r = 0.341, P = 0.022, respectively) was not as powerful as that found with TLR2, which may indicate that TLR7 upregulation is not necessarily related to increased signaling towards NF-κB activation.

As for the correlation with the miRNAs, miR-99b and miR-125b showed a significant negative correlation with TLR7 levels (Figures 7C and 7D), in agreement with what was observed for TLR2. Again, no relationship was observed for miR-125a; however, there was a trend towards a negative correlation too (Figure S2B). Although these data might be an indirect consequence of the strong correlation between TLR2 and TLR7, results support the idea that the expression of the cluster is related to the TLR/NF-κB pathway. Moreover, the expression of the miR-99b/let-7e/miR-125a cluster might be linked to that of the paralogous cluster miR-125b/miR-99a/let-7c through the TLR/NF-κB pathway.

**Figure 8:**
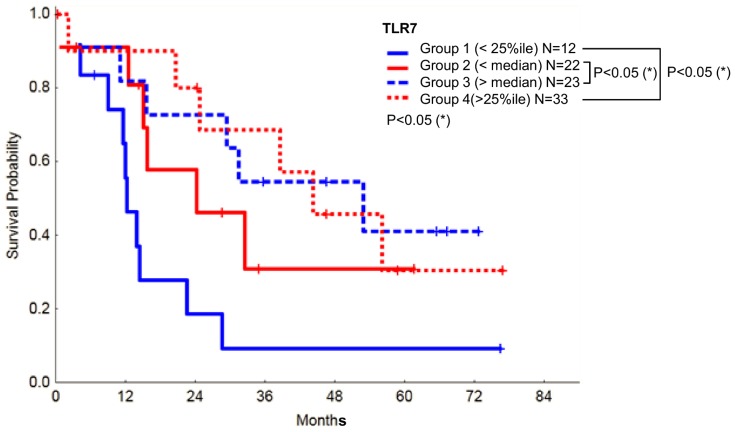
Correlation between the relative expression of TLR7 and overall survival in MDS. Levels of TLR7 are directly correlated with patient survival.

To confirm the relevance of TLR7 in MDS we also performed a survival analysis in MDS patients with high or low levels of this receptor. Of interest, high TLR7 levels appeared to be beneficial for overall survival of the patients (Figure 8), in contrast with data obtained for TLR2 [16].

### High endogenous levels of miR-125a prevent TLR-induced NF-κB activation

As discussed above, the reverse correlation between miR-99b and the expression of the TLR pathway members, along with the fact that the combination of ectopic miR-99b and miR-125a activates NF-κB, lead us to hypothesize that the deregulation of the miR-99b/let-7e/miR-125a cluster and aberrations in the TLR pathway could be mutually exclusive mechanisms that release selective pressure towards each other. However, because miR-125a levels did not correlate with the expression of TLRs and their downstream genes, we reasoned that the expression of this miRNA could be elevated also in cells with hyperactivated TLR signaling. Therefore, our next step was to elucidate the role of endogenous miR-125a on NF-κB activation in the presence of TLR signaling.

First, we screened several human acute myeloid leukemia (AML) cell lines for miR-125a expression in order to find a good model in which miR-125a levels were high. AML cell lines were utilized due to the lack of an easily transfectable MDS cell line. The AML myeloblastic cell line KG1 was selected for the experiments for having the most similar miRNA expression pattern to MDS cells (Figure S3) and because MyD88 is overexpressed in this cell line [15], which guarantees a high TLR activity. To explore the effect of endogenous miR-125a on NF-κB activity, KG1 cells were transfected with the same reporter vector used before and treated with an ASO for miR-125a to specifically inhibit the expression and functionality of this miRNA. To reproduce the conditions of TLR activation, the TLR/NF-κB pathway was simultaneously stimulated with the specific TLR4/TLR2 and TLR2 agonists LPS and PAM3, respectively.

**Figure 9:**
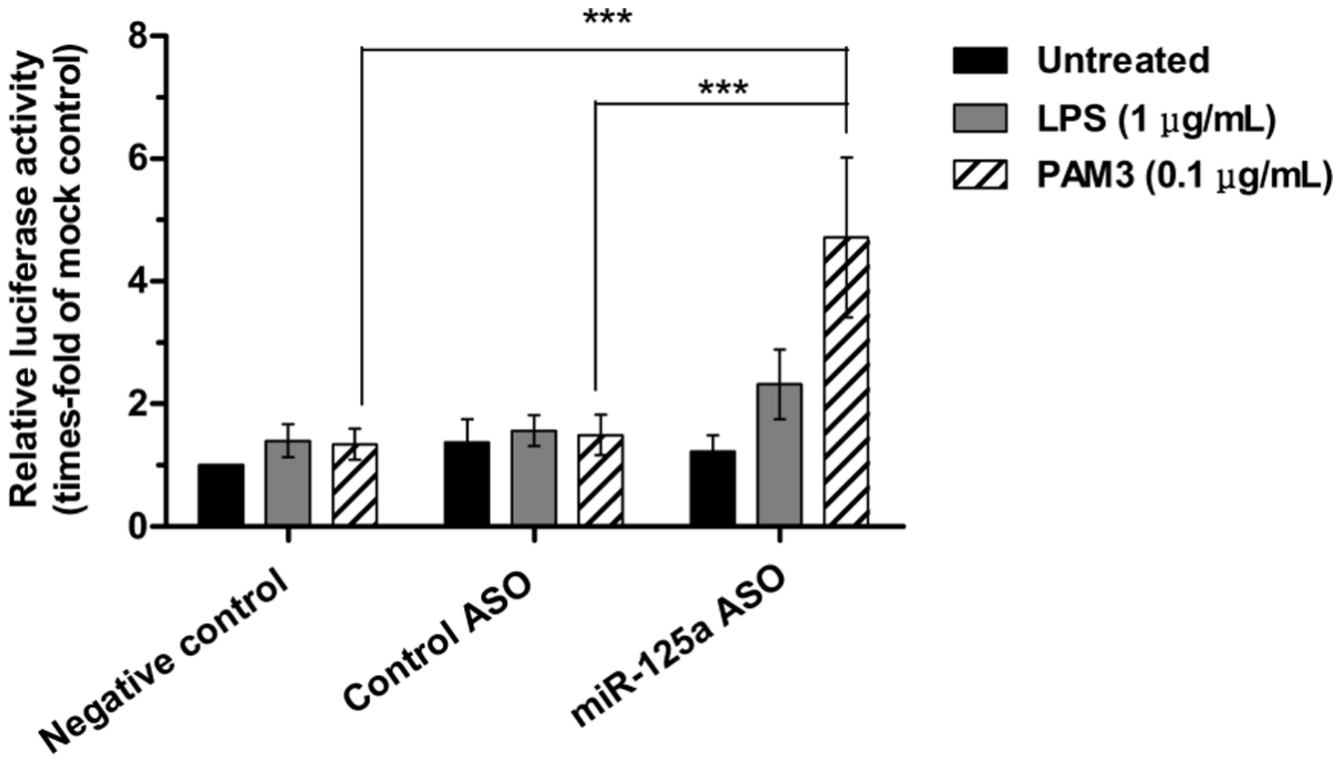
Effect of TLR stimulation and miR-125a inhibition on NF-κB activity in KG1 cells. NF-κB activation was measured after 24 hours from nucleofection of KG1 cells with the luciferase vectors and treatment with ASO. Results are expressed as relative to cells transfected with mock and represent mean ± SEM of n = 4 independent experiments. Method disclosure: technical problems regarding the endogenous *Renilla* control were experienced during these luciferase assays; only one experiment out of four efficiently expressed the *Renilla* luciferase and could be properly normalized. Because normalized results were almost identical to non-normalized data, we conducted a joint statistical analysis of the four experiments. Statistical significance: ***P<0.001.

As determined by luciferase reporter assay, miR-125a inhibition did not have a significant effect on NF-κB activity in basal conditions. However, if cells were simultaneously stimulated with TLR agonists, miR-125a inhibition resulted in the enhancement of NF-κB activation (Figure 9). Importantly, this synergy was especially strong in the case of TLR2-specific stimulation, pointing out the importance of miR-125a in the modulation of the effects of this receptor and its downstream effectors.

These results contradict our hypothesis that miR-125a collaborates with the TLR pathways on NF-κB activation and unveil a potential inhibitory activity of this miRNA in the presence of TLR signaling. This blockade of TLR-induced NF-κB activity could occur through the repression of one or more TLR adaptors. Herein, we suggest TRAF6 as a potential target because it has been postulated that this molecule is tightly regulated by a miRNA feedback loop in hematopoietic progenitors and stem cells [8] and, importantly, the 3′ UTR of its mRNA contains a conserved miR-125a binding site [63]. It is also possible that miR-125a inhibits the expression of the NF-κB activating kinase IKKγ (NEMO), which was predicted as a target of this miRNA by miRGen [64]. The implications of the dual role of miR-125a on NF-κB activity will be further discussed below.

### miR-125a inhibition in K562 cells favors Ara-C-induced erythroid differentiation

In two independent studies of miRNA signatures in AML, miR-125a was found to be downregulated in AML blasts as compared with normal CD34^+^ cells [65], [66]. It was suggested that this might not be a pathological but a differentiation-related event, attributed to the natural loss of expression of this miRNA in more differentiated cells [29]. The notion that miR-125a expression could be gradually lost during commitment, along with the reported involvement of this miRNA in the maintenance of hematopoietic stem cell number [34], [36] and its differential overexpression in patient BM suggested by our previous results, lead us to hypothesize that miR-125a could be responsible for some of the differentiation abnormalities characteristic of MDS. Thus, we next focused on the effects of this miRNA on hematopoietic differentiation.

Our group has recently demonstrated that MDS CD34^+^ primary cells can differentiate towards the erythroid lineage [15]. To explore the potential role of miR-125a on differentiation, we first studied its effects on the erythroleukemia cell line K562, which undergoes erythroid differentiation upon stimulation with several agents, such as Ara-C [67]. Prior to performing colony formation assays for the differentiation study, the effects of miR-125a ASO on cell proliferation and viability were tested in order to avoid changes in colony number caused by the cytotoxicity of the treatment. Treatment for 48 hours with ASO at an effective dose (1 µM) demonstrated to be safe in K562 cells (Figures S4A–C); however a significant unspecific inhibition of miR-125b (which shares the seed sequence with miR-125a) was detected (Figure S4D). For this reason, results presented herein could also be attributed, at least in part, to the inhibition of miR-125b. Nevertheless, the inhibition of miR-125a at the same dose was clearly stronger (Figure S4A).

**Figure 10:**
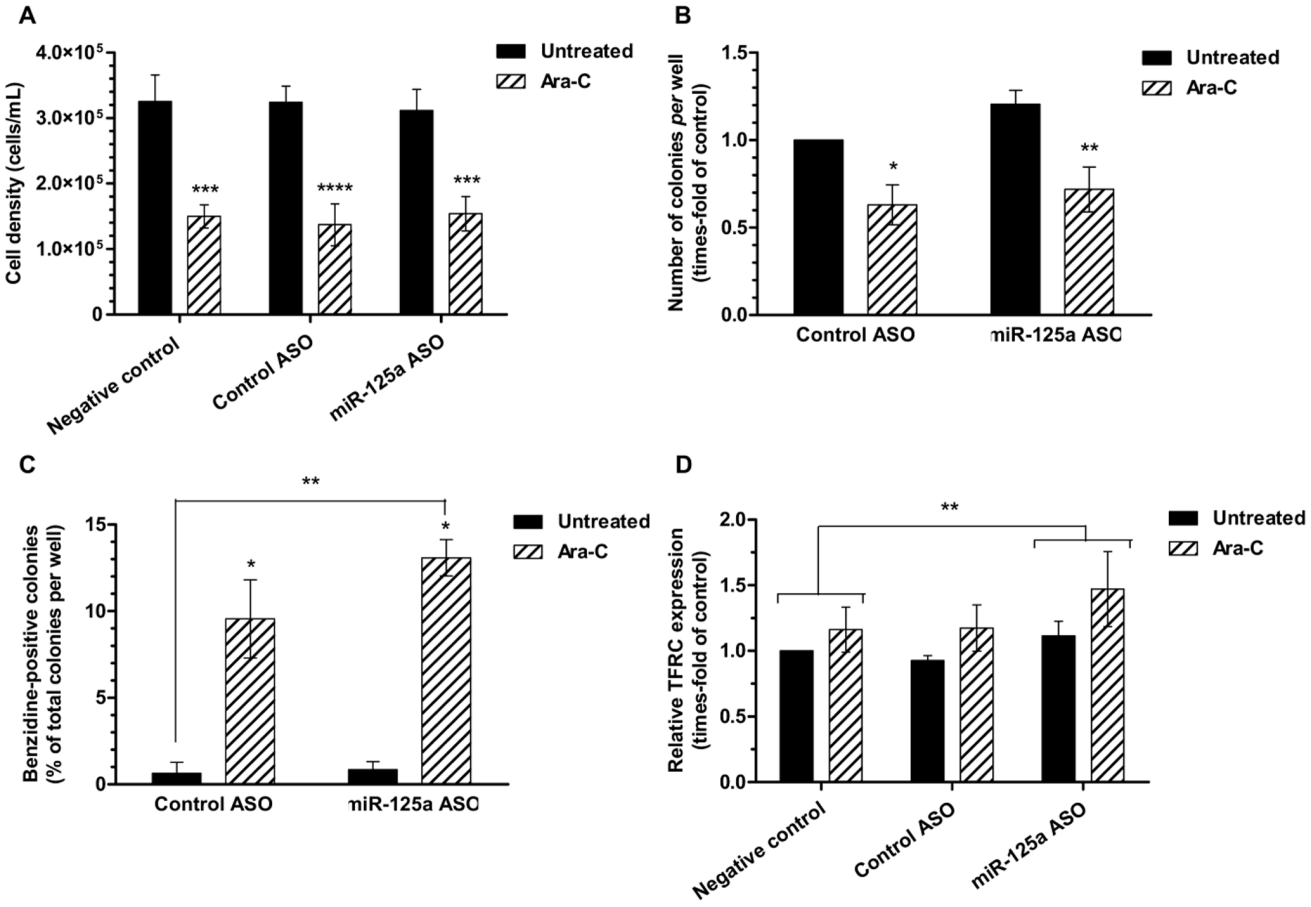
Effect of miR-125a inhibition on Ara-C-stimulated erythroid differentiation of K562 cells. K562 cells pre-treated with 1 µM ASO for 6 hours and treated with ASO for 48 hours were plated in MC for 4 days before being counted and collected for the corresponding assays. (**A**) Effect on cell density after 48 hours (n = 5). (**B**) Colony formation assay (n = 5). (**C**) Benzidine-positive colony count (n = 3). The proportion of erythroid-like colonies after 4 days from plating is expressed as the number of benzidine-positive colonies, normalized to the total colony number. (**D**) Expression of CD71 (TFRC) in K562 colonies. Statistical analysis represents a grouped analysis of the main effect of ASO (n = 4). (**A–D**) Data represent mean ± SEM. Statistical significance: *P<0.05; **P<0.01; ***P<0.001; ****P<0.0001.

Treatment of K562 cells with 1 µM Ara-C induced a significant (P<0.001) decrease on cell number after 48 hours (Figure 10A) without affecting viability (data not shown), suggesting the induction of a proliferative arrest. No differences in cell number or viability were found when cells were co-treated with ASO. As expected, after 4 days of incubation of colonies, Ara-C reduced colony number (Figure 10B) while it increased the number of benzidine-positive (heme group-forming or erythroid-like) colonies (Figure 10C). miR-125a inhibition alone did not induce significant changes in colony number or benzidine-positive K562 cells; however, when combined with Ara-C, it increased the number of erythroid-like colonies (Figures 10B–C). This suggests that miR-125a inhibition does not trigger differentiation *per se* but favors differentiation started by other stimulus. Thus, endogenous miR-125a might partially block or inhibit erythroid differentiation.

In order to confirm the effects of miR-125a on Ara-C-induced differentiation in K562 cells, we also analyzed the expression of the common erythroid markers glycophorin A (GYPA), erythropoietin receptor (EPO-R), and CD71/TFRC. Levels of EPO-R and GYPA mRNA (mature erythroid markers) increased with the treatment with Ara-C but did not show any changes upon co-treatment with miR-125a ASO (results not shown), while the early marker CD71 was significantly higher in all ASO-treated cells (Figure 10D), confirming that miR-125a inhibition favors Ara-C-stimulated erythroid differentiation in K562 cells.

### MDS-L cells express high levels of miR-125a/miR-125b

**Figure 11:**
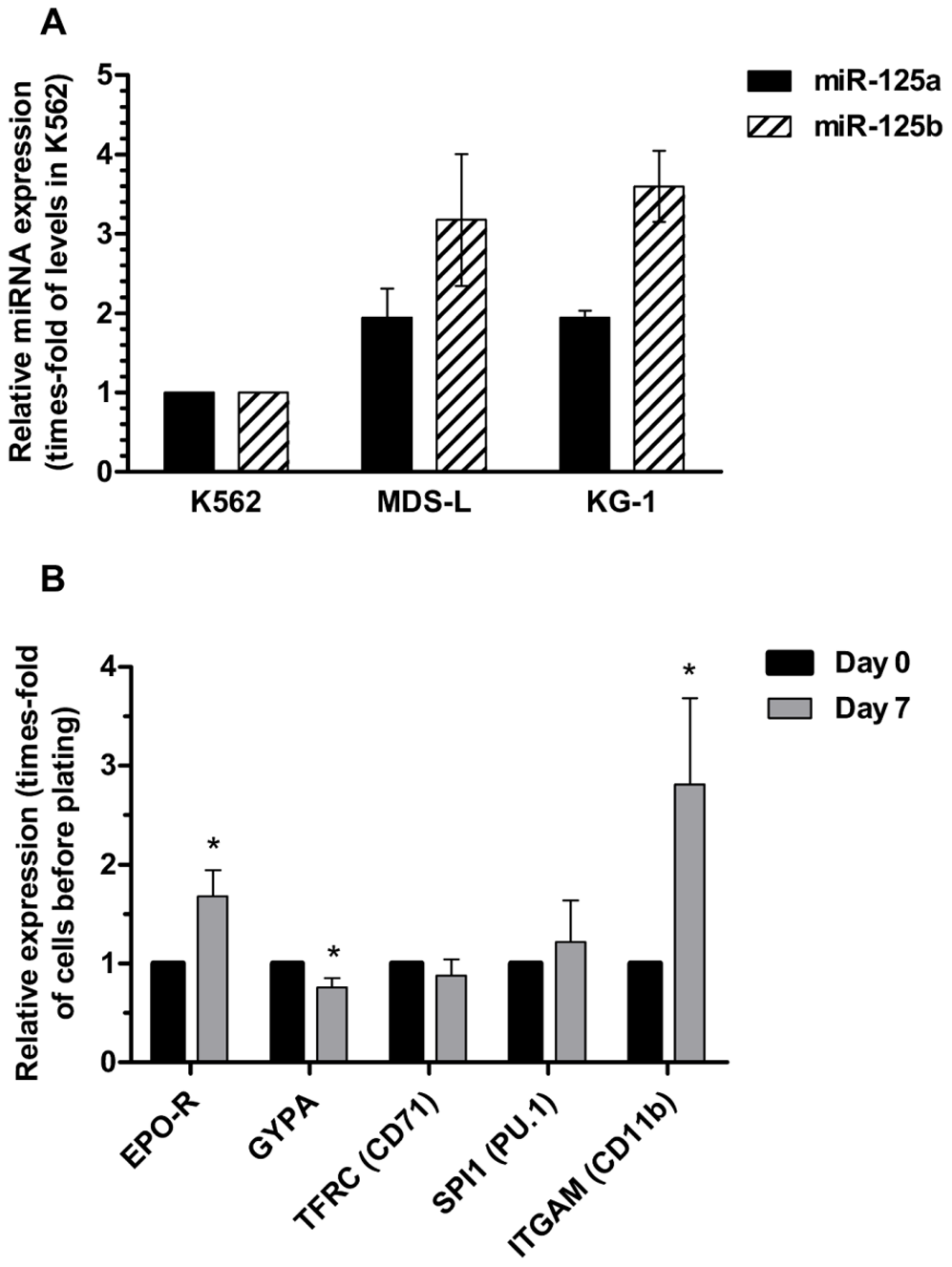
Characterization of MDS-L cells. (**A**) Basal expression levels of miR-125a and miR-125b in myeloid leukemia and MDS cell lines. miRNA levels are represented as the relative value to expression in K562 cells. Expression of miR-125a in MDS-L cells was 5–10 times higher than that of miR-125b (note that relative levels of miR-125a were calculated independently from those of miR-125b). Data represent mean ± SEM of n = 4 independent experiments. (**B**) Spontaneous differentiation of MDS-L cells after a 7-day culture in methylcellulose, expressed as the relative expression of differentiation markers, assessed by qPCR in MDS-L colonies collected after 7 days of incubation in methylcellulose. Data represent mean ± SEM of n = 3 independent experiments. Statistical significance: *P<0.05.

We then sought to confirm the effects of miR-125a on differentiation using a model that was more representative of MDS. For this purpose, we utilized the human cell line MDS-L, which has been established from an MDS patient [49]. We first analyzed miR-125a and miR-125b levels in this cell line and compared them to those of K562 cells. As shown in Figure 11A, miR-125a and miR-125b expression in MDS-L cells was higher than in K562 cells and similar to that in KG1 cells, which agrees with overexpression in MDS patients and allows the study by inhibition of the miRNA with ASO.

Cytotoxicity and effectiveness of the ASOs, as well as their specificity, were tested prior to proceeding to the colony formation assays. Treatment with 1 µM ASO inhibited approximately 80% of the relative expression of miR-125a and did not affect cell density or viability (Figure S5A,C–D). Unspecific inhibition of miR-125b was not statistically significant in this cell line but should also be taken into account (Figure S5B).

### MDS-L cells can spontaneously differentiate in methoculture

Similarly to what was observed on K562 cells, miR-125a inhibition alone did not significantly affect colony formation ability of MDS-L cells or the expression of any of the erythroid markers of study (data not shown). Moreover, levels of the myeloid markers PU.1 (SPI1) and integrin γ-M (ITGAM, or CD11b) were also determined by qPCR and no significant changes were detected, although there was a trend towards an increase in CD11b levels (Figure S6A–E).

We plated untreated cells and allowed them to grow for one week in order to determine if they would undergo spontaneous differentiation upon stimulation with the growth factors present in the methylcellulose medium. After this time, levels of the differentiation markers studied before were analyzed by qPCR (Figure 11B). Interestingly, colonies were significantly enriched on CD11b, a mature myeloid marker, suggesting that untreated MDS-L cells can undergo certain degree of spontaneous myeloid differentiation in methoculture. Surprisingly, cells also underwent significant changes in the expression of the erythroid markers EPO-R and GYPA, which increased and decreased, respectively. It is therefore not clear if MDS-L cells acquire a myeloid phenotype in methylcellulose cultures. However, these spontaneous changes in the expression of various differentiation markers could be the reason why the inhibition of miR-125a alone did not result in any observable change in the differentiation state of these cells.

### miR-125a inhibition enhances erythroid differentiation induced by MyD88 inhibition in MDS-L cells

Because we could not address the effects of miR-125a on differentiation in untreated MDS-L cells, we sought to study the activity of this miRNA in the presence of a differentiating stimulus, following the same approach used with K562 cells. Our group recently showed that inhibition of MyD88 induces erythroid differentiation in MDS CD34^+^ primary cells [15]. For this reason, we decided to examine differentiation of MDS-L cells in the presence of a specific inhibitor of MyD88.

**Figure 12:**
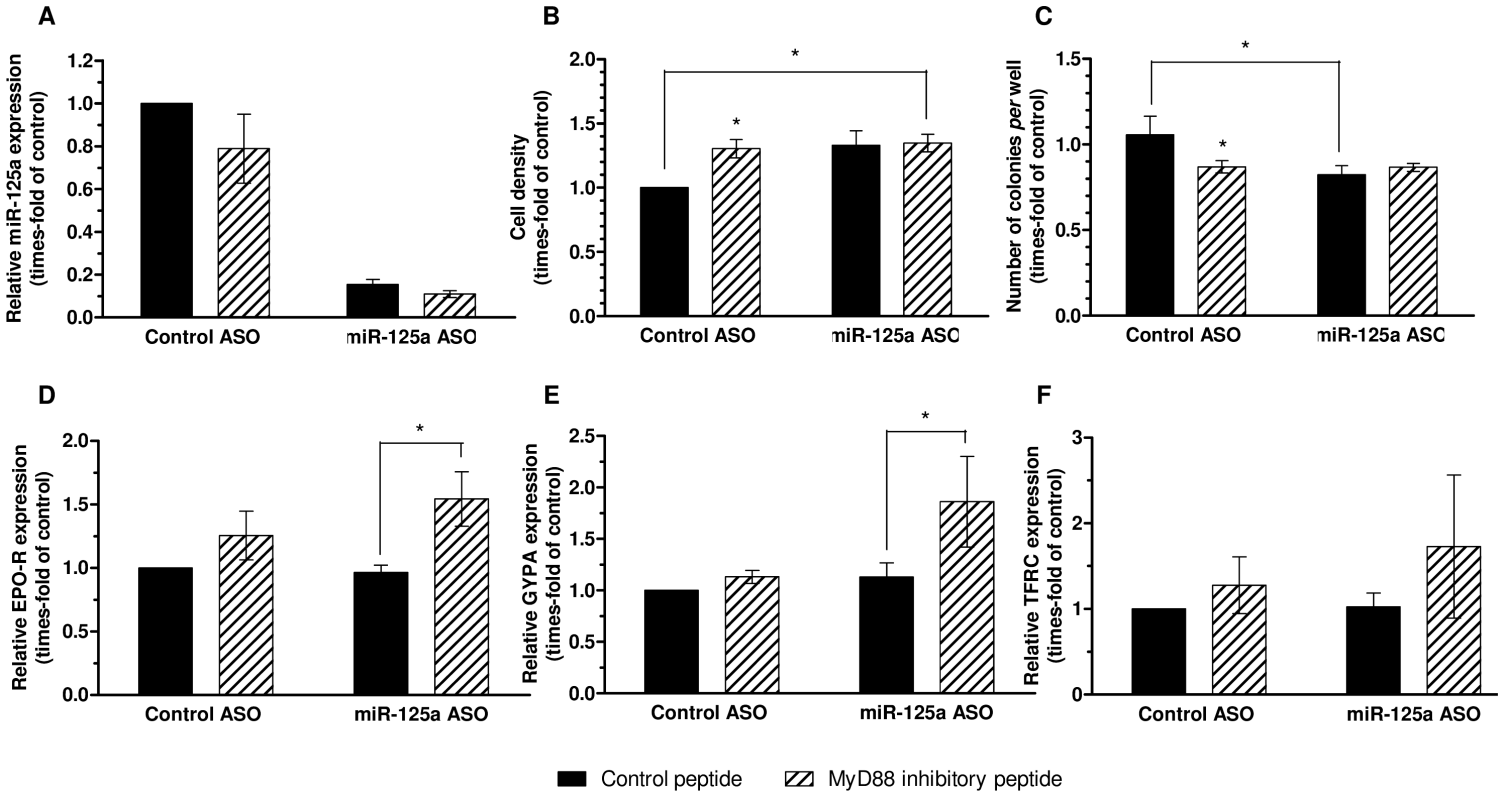
Effect of the inhibition of miR-125a and TLR2-NF-κB pathway in MDS-L cells. Black bars represent cells treated with 5 µM control peptide, and striped bars represent cells treated with 5 µM MyD88-inhibitor peptide. (**A**) Effectiveness of miR-125a ASO in MDS-L cells, expressed as relative miR-125a expression levels after 48 hours of treatment with 1 µM ASO. (**B**) Cell density after 48 hours of treatment. (**C**) Colony formation assay after 7 days from plating in methylcellulose. (**D–F**) Relative expression of EPO-R, GYPA and CD71 (TFRC), respectively, measured by qPCR after a 7-day methylcellulose culture of cells previously treated for 48 hours with 1 µM ASOs. (**A–F**) Data represent mean ± SEM of n = 3 independent experiments. Statistical significance: *P<0.05.

Interestingly, MyD88 inhibition itself slightly decreased miR-125a levels after 48 hours of treatment (Figure 12A), which could be again indicative of a link between miR-125a regulation and the TLR/NF-κB pathway. The MyD88-inhibitor peptide and miR-125a ASO slightly increased cell number after 48 hours of treatment, both separately and in combination (Figure 12B). Similarly, both treatments induced a mild but significant decrease in colony number (Figure 12C) in methocultures. These effects on the proliferative activity and colony formation of MDS-L cells were quite moderate. The analysis of the expression of differentiation markers, however, revealed that miR-125a inhibition potentiates differentiation of MDS-L cells towards the erythroid lineage (Figures 12D–F). Accordingly, no differences were detected in the expression of myeloid markers (Figure S7).

These results confirm that endogenous miR-125a interferes with erythroid differentiation. In agreement with this finding, the integrated database miRGen [64] revealed that the erythroid differentiation-associated factors ETS1 and ERAF (α-hemoglobin stabilizing protein, AHSP) are potential target genes of this miRNA. Particularly, inhibition of ETS1 expression could be a possible mechanism for the repression of erythroid differentiation observed under our experimental conditions.

Because our results pointed to a decrease in erythroid differentiation in MDS patients overexpressing miR-125a, we searched for signs of erythroid hypoplasia in this subset of patients. Although only 7 out of 48 patients (15%) presented with erythroid hypoplasia, all of them had miR-125a expression levels over 3-fold of controls, being 4 of those patients at the 50^th^ percentile rank, and 2 others at the 75^th^ percentile rank. That is, erythroid hypoplasia was only observed in patients with considerably high miR-125a levels. However, these data are too preliminary and the association is not strong enough to establish a causal relationship between miR-125a overexpression and decreased erythroid differentiation in MDS patients. Furthermore, we believe that erythroid commitment may not be the only process of hematopoiesis which is modulated by this miRNA. Interestingly, our bioinformatic search also predicted a number of positive regulators of myeloid differentiation as potential targets of miR-125a, including core-binding factor β (CBFB) and SPI1 (PU.1). This suggests that this miRNA could participate in the fine-tuning of hematopoietic differentiation by modulating the expression of genes involved in differentiation through more than one lineage. Because “normal” commitment requires synchronized changes in the expression of these and other hematopoietic regulators, sustained overexpression of miR-125a might result in the deregulation or dyssynchrony of hematopoiesis rather than in the arrest of differentiation along just one lineage. Taken together, these results indicate that the aberrant expression of miR-125a in BM cells could play an important role in hematopoietic aberrations characteristic of MDS.

## Discussion

In the present work we aimed to study the involvement of the homologous miRNAs miR-125a and miR-125b in the pathogenesis of MDS and their possible participation in the regulation of innate immunity pathways and the modulation of hematopoietic differentiation. Our results indicate that both miRNAs are overexpressed in BM CD34^+^ cells of MDS patients and that their levels are correlated, which may indicate that they share common mechanisms of regulation that could be altered in the pathogenesis of this disease. However, miR-125a was expressed in significantly higher levels than miR-125b, both in MDS patients and in healthy donors. This suggests that, despite miR-125b having been more deeply studied in hematopoiesis and hematopoietic malignancies [35], [37], [38], [40], [42], miR-125a may in fact play a more important role, at least in MDS. For instance, high miR-125a levels were found to be directly correlated with prognosis, while no correlation was found for miR-125b levels. These results confirm the relevance of miR-125a in this group of diseases and fully agree with two recently published works in which constitutive expression of miR-125a in BM transplanted into irradiated mice induced various phenotypes indicative of myeloproliferative neoplasms [36], [41]. Importantly, in one of those studies [41], miR-125b was dismissed because it induced weaker phenotypes than miR-125a, supporting our conclusion that miR-125b is not as relevant as miR-125a in MDS.

The expression of the other members of the miR-99b/let-7e/miR-125a cluster was also found to be strongly correlated to that of miR-125a but, similarly to miR-125b, no statistically significant differences were found between MDS patients and healthy controls and their total levels were also considerably lower. Of note, let-7e had previously been identified as a miRNA which is differentially expressed in MDS [33]. Although we found evidence of a coordinated regulation of miR-125b and the three components of the miR-99b/let-7e/miR-125a cluster, our results suggest that mature miR-125a undergoes alternative mechanisms of regulation which are independent of other members of its genomic cluster and the homologous cluster miR-99a/let-7c/miR-125b. On one hand, the higher levels of miR-125a in comparison to the other related miRNAs might be explained by increased transcription, which could occur independently of the rest of the cluster since miR-125a is localized at the 3′ end (Figure S1). For instance, conserved binding sites for various transcription factors, which could be responsible for the independent transcription of miR-125a, exist in the intergenic region between miR-99b and let-7e [68]. On the other hand, provided that the association between the expression levels of the three components of the cluster is rather strong, it is more likely that they are transcribed together, giving rise to a polycistronic product. Cluster-independent expression could be explained by a reduced decay (higher stability) of miR-125a or by changes in processing of the primary transcript during miRNA biogenesis, which we believe is the most reasonable explanation. It is possible therefore to hypothesize that miR-125a can be independently or preferentially expressed under certain stimuli to carry out alternative functions.

Owing to the strong correlation between miR-125a and MDS survival, we next studied more deeply the mechanisms through which this miRNA could be participating in the pathogenesis and/or progression of the disease. Two important functions were explored: its regulatory role in the TLR/MyD88/NF-κB pathway and its participation in the modulation of hematopoietic differentiation.

**Figure 13:**
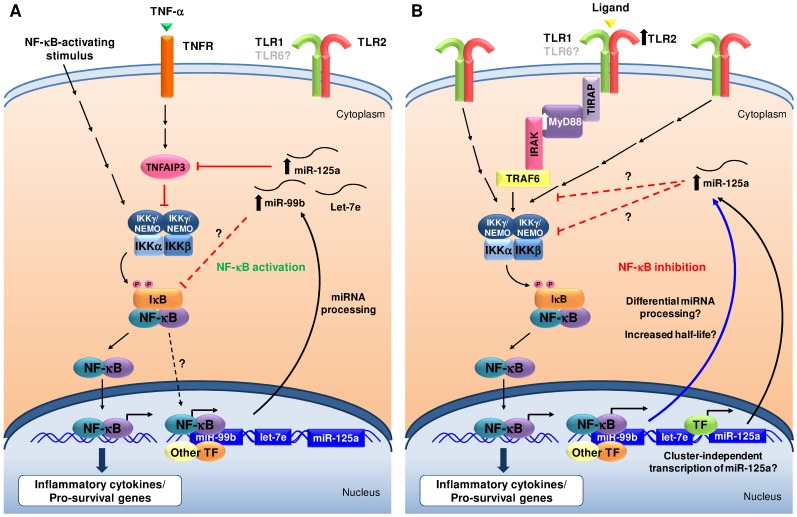
Proposed model for miR-125a regulation in MDS cells. Discontinuous arrows represent speculations and unknown mechanisms; consecutive arrows represent known pathways that do not need to be explained for the understanding of the figure. (**A**) In the absence of TLR signaling, the miR-99b/let-7e/miR-125a cluster (and very likely also miR-125b) is coordinately upregulated, via transcriptional activation by either NF-κB as a part of a positive feedback loop or by other transcription factors (TF). miR-125a, presumably in collaboration with miR-99b, enhances NF-κB activation, probably through the inhibition of the TNF-induced NF-κB inhibitor TNFAIP3 [43] and/or other inhibitors, such as IκBε. Thus, the expression of the miR-99b/let-7e/miR-125a cluster may favor survival of hematopoietic cells, protecting them from the deadly effects of TNF-α. (**B**) Upon TLR signaling, NF-κB is activated through a cascade of adaptor proteins. Under these conditions, the miR-99b/let-7e/miR-125a cluster is not expressed (or expressed at low levels) but the expression of miR-125a might be independently induced by unknown (maybe disease-related) mechanisms, such as the initiation of transcription at alternative promoter regions or, more likely, the differential processing of the primary transcript in one or more stages of miRNA biogenesis. Highly increased levels of miR-125a could preferentially target the mRNA of one or more genes downstream of TLRs and participate in the negative modulation of proinflammatory signaling. In MDS, elevated miR-125a levels in cells with normal TLR signaling (A) would favor sustained NF-κB activation and pro-survival effects; while in those cells with increased TLR/MyD88 levels and activation (B), high miR-125a levels would negatively modulate NF-κB activation. However, in this case, the hyperactivation of the TLR/MyD88/NF-κB pathway would probably mask the inhibitory effects of miR-125a. Additionally, in both cases, (A) and (B), overexpression of miR-125a in MDS patients would favor aberrant differentiation so its effects in either case would be detrimental for the course of the disease.

As for the regulation of the TLR/MyD88/NF-κB axis, we found opposing activities of miR-125a on NF-κB activation under different activation states of the pathway. This duality seems contradictory and needs a cautious interpretation because the apparently different effects of miR-125a may respond to different molecular mechanisms active in the cell lines utilized. However, it can be hypothesized that the dual role of this miRNA in NF-κB modulation is a fine-tuning mechanism which switches from activation to repression upon stimulation of TLR signaling. A similar double role in NF-κB activation has been described for miR-155, which is upregulated by TLR signaling during the macrophage inflammatory response to infection and inhibits the activation of inflammatory pathways in myeloid cells (reviewed in [17]). Ectopic expression of miR-155 in unstimulated BM cells, however, leads to increased inflammation, mimicking TLR stimulation and inducing a myeloproliferative phenotype [69]. Notably, miR-155 is also involved in the regulation of hematopoiesis and has been reported to be overexpressed in hematologic tumors, similarly to miR-125a [70]. Furthermore, the hypothesis of the dual activity would explain the two types of regulation of miR-125a expression (cluster-associated and cluster-independent) reported in this work. Herein, we propose a model in which miR-125a activates or inhibits NF-κB depending on the concurrent activation of TLR signaling and the co-expression of other cluster members. This model is depicted in Figure 13.

Although we did not further explore the mechanisms of activation or inhibition of the NF-κB pathway, the model proposed points to an important role for miR-125a in the modulation of NF-κB activation, as it was suggested before for this miRNA [43]. In patients without alterations in the TLR pathway, overexpressed miR-125a might enhance NF-κB activation; whereas in those patients with hyperactivated TLR pathway, miR-125a would counteract the activation of NF-κB. However, this effect could be masked by the strong overexpression of the members of the pathway and the continuous stimulation of the signal by positive feedback loops [14], [16]. Thus, NF-κB signaling would be constitutively activated in patients with and without TLR/MyD88 hyperactivation.

Nevertheless, it has been postulated that NF-κB activation in CD34^+^ cells is not enough to induce changes in stem and progenitor cell growth and differentiation [71]. Because there is evidence of miR-125a playing a role in the maintenance and self-renewal of the hematopoietic stem/progenitor state in normal BM cells [34]–[36], we hypothesized that this miRNA contributes to MDS in other ways in addition to NF-κB modulation. Our results showed that the inhibition of miR-125a in K562 and MDS-L cells enhances erythroid differentiation induced by Ara-C and a MyD88 inhibitor, respectively. These results suggest that miR-125a inhibits or interferes with erythroid differentiation and that its overexpression in MDS patients might contribute to the loss of fine-tuning of hematopoiesis. Furthermore, the results of a bioinformatic search for predicted targets revealed that miR-125a may not only interfere with erythroid differentiation but also with certain stages of granulocytic/monocytic or megakaryocytic differentiation, supporting the idea that miR-125a overexpression participates in the pathogenesis of MDS by inducing an overall deregulation of hematopoiesis and the appearance of subsequent hematopoietic aberrations. Nevertheless, it needs to be kept in mind that miR-125b was also partially inhibited by miR-125a ASO in our differentiation experiments, so we cannot rule out the cooperation or redundancy of function of this miRNA in the regulation of hematopoiesis in MDS.

In summary, this is, to the best of our knowledge, the first time that miR-125a levels have been reported to be inversely connected to survival in MDS. Overall, our results indicate that the deregulation of miR-125a expression may contribute in various ways to the disease and that this molecule is a therapeutic target of interest that should be further explored. Moreover, miR-125a is a potential prognosis marker of great utility in the clinical practice, since miRNAs, including miR-125a, miR-99b and let-7e, have been reported to be secreted to peripheral blood and serve as circulating disease biomarkers [30], [72]–[75].

Last, we have found that TLR7 is not only overexpressed in MDS and strongly correlated with TLR2 expression, but also that it is significantly correlated with a better prognosis. It is unclear how TLR7 may contribute to patient survival in MDS, but the mechanism may be related to the fact that its stimulation appears to induce myeloid differentiation in CD34^+^ progenitors [9]. The direct correlation between the expression levels of TLR7 and TLR2 suggests that they share regulatory mechanisms or that the expression of TLR7 is indirectly induced by TLR2, which could occur via NF-κB activation [76]. Because TLR7 recognizes single stranded RNA molecules [77], it is also possible that its activation is triggered by miRNAs in response to TLR2 stimulation. For instance, it has been recently reported that TLR7/TLR8 can be activated by secreted miRNAs. Importantly, TLR7 activation by those miRNAs did not result in NF-κB activation [46], although this is the typical consequence of TLR7 stimulation [78]–[80]. To sum up, our results confirm that innate immunity signaling is involved in the pathogenesis and/or progression of MDS and suggest that TLR7 could be a good prognosis marker in this group of diseases.

## Supporting Information

Figure S1 **miR-125a cluster in humans.** miR-125a, miR-99b and let-7e are clustered together in a ∼750 bp intergenic region located in chromosome 19q13.41. NR: National Center for Biotechnology Information (NCBI) Reference sequence number.
(TIF)

Figure S2 **(A) Correlation between the relative expression of JMJD3 and miR-99b in MDS CD34^+^ cells.** Eight outliers removed by ROUT method. **(B) Correlation between the relative expression of TLR7 and miR-125a in MDS CD34^+^ cells.** Seven outliers removed by ROUT method. (**A**) **and** (**B**) “Low” and “high” expression cohorts were established based on comparison with the mean relative expression value.
(TIF)

Figure S3 **Basal expression levels of miR-125a and miR-125b in AML cell lines.** miRNA levels are represented as the relative value to expression in THP-1 cells (note that relative levels of miR-125a were calculated independently of those of miR-125b). Data represent the mean ± SEM of n = 3.
(TIF)

Figure S4 **Efficiency of miR-125a inhibition in K562 cells.** (**A**) Dose-response of miR-125a ASO. K562 cells were treated with increasing doses of ASO (100–1000 nM) for 48 hours and miR-125a expression was assessed by qPCR (n>2). (**B–C**) Effect of miR-125a inhibition on cell density and viability, respectively, of K562 cells after 48 hours of treatment with 1 µM ASO (n = 6). Negative controls are untreated cells. (**D**) Unspecific effect of miR-125a inhibition on miR-125b expression in K562 cells. (**A–D**) Data represent mean ± SEM. Statistical significance: *P<0.05; ***P<0.001; ****P<0.0001.
(TIF)

Figure S5 **Efficiency of miR-125a inhibition in MDS-L cells.** Cells were treated with 1 µM control and miR-125a ASO for 48 hours. (**A**) Changes in relative miR-125a expression, determined by qPCR. (**B**) Unspecific effect of miR-125a inhibition on miR-125b expression in MDS-L cells. (**C–D**) Effect of miR-125a inhibition on cell density and viability, respectively. (**A–D**) Data represent mean ± SEM of n = 3 experiments. Statistical significance: ****P<0.0001.
(TIF)

Figure S6 **Effect of miR-125a inhibition on MDS-L cells differentiation.** Relative expression levels of the differentiation markers (A) EPO-R, (B) GYPA, (C) CD71, (D) PU.1, (E) CD11b were determined by qPCR in 7-day colony samples previously treated for 48 hours with 1 µM ASOs. Data represent mean ± SEM of n = 8 independent experiments.
(TIF)

Figure S7 **Effect of the inhibition of miR-125a and TLR2-NF-κB pathway in MDS-L cells.** Relative expression levels of the myeloid differentiation markers (**A**) PU.1 and (**B**) CD11b were measured in colony samples by qPCR after a 7-day methylcellulose culture of cells previously treated with 1 µM ASO and 5 µM of the corresponding peptide. Black bars represent cells treated with control peptide, and striped bars represent cells treated with MyD88 inhibitor peptide.
(TIF)

Table S1 Patient characteristics.
(TIF)

Table S2 Sequences of anti-sense oligonucleotides used for miR-125a inhibition assays. (m) = 2′O-methyl modification; (*) = phosphotiorate bond; (3′-Chl) = 3′ Cholesterol modification.
(TIF)
