# Supplementary figures and images for: Overexpression of miR-125a in Myelodysplastic Syndrome CD34+ Cells Modulates NF-κB Activation and Enhances Erythroid Differentiation Arrest
Source: PLoS One. 2014 Apr 1;9(4):e93404. doi: 10.1371/journal.pone.0093404 (PMC3972113; doi:10.1371/journal.pone.0093404)

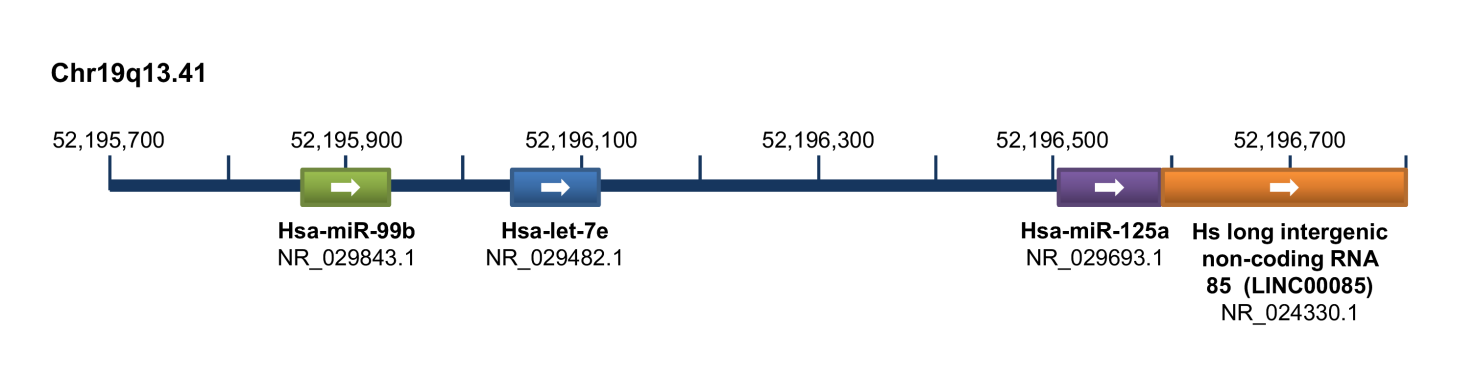

Supplement: Figure S1 — miR-125a cluster in humans. miR-125a, miR-99b and let-7e are clustered together in a ∼750 bp intergenic region located in chromosome 19q13.41. NR: National Center for Biotechnology Information (NCBI) Reference sequence number. (TIF) [file pone.0093404.s001.tif]

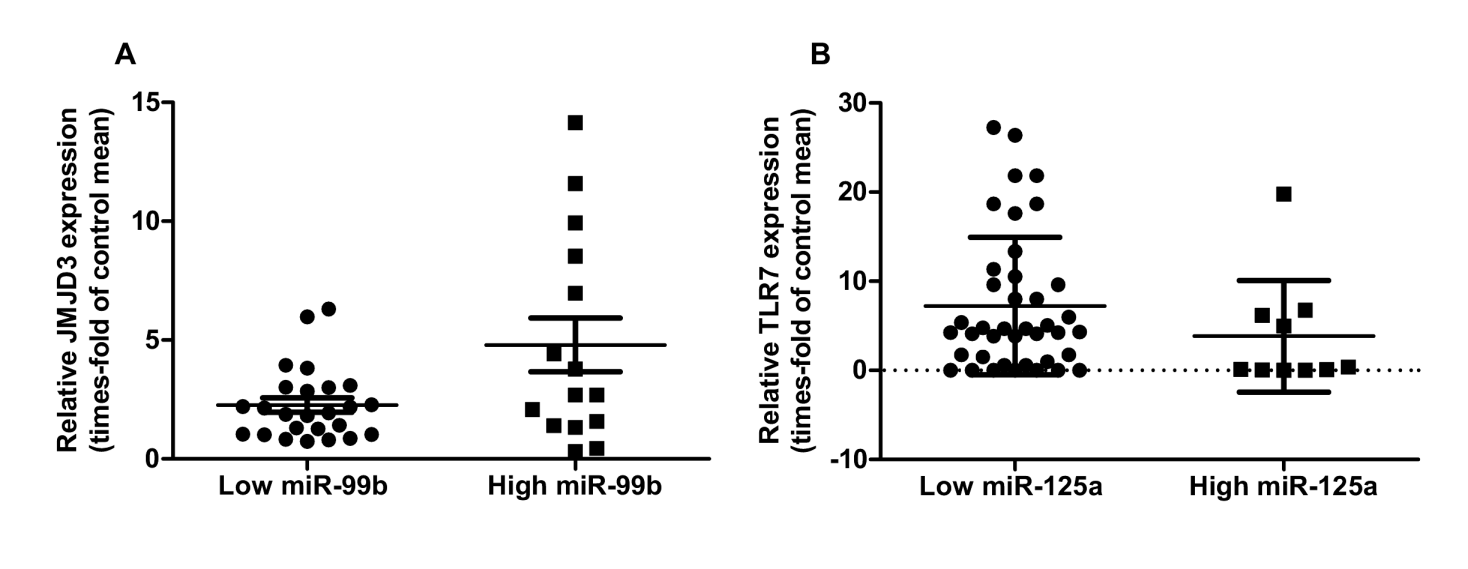

Supplement: Figure S2 — (A) Correlation between the relative expression of JMJD3 and miR-99b in MDS CD34+ cells. Eight outliers removed by ROUT method. (B) Correlation between the relative expression of TLR7 and miR-125a in MDS CD34+ cells. Seven outliers removed by ROUT method. (A) and (B) “Low” and “high” expression cohorts were established based on comparison with the mean relative expression value. (TIF) [file pone.0093404.s002.tif]

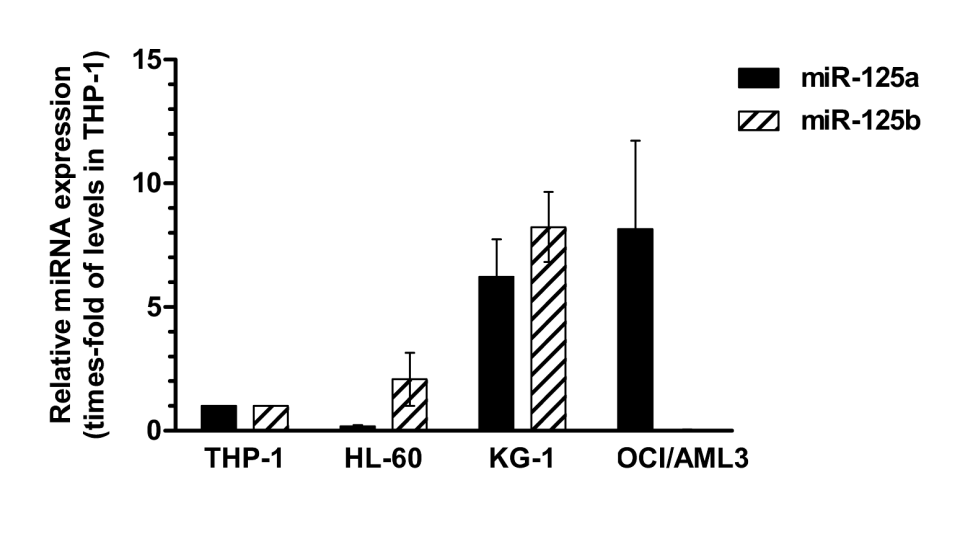

Supplement: Figure S3 — Basal expression levels of miR-125a and miR-125b in AML cell lines. miRNA levels are represented as the relative value to expression in THP-1 cells (note that relative levels of miR-125a were calculated independently of those of miR-125b). Data represent the mean ± SEM of n = 3. (TIF) [file pone.0093404.s003.tif]

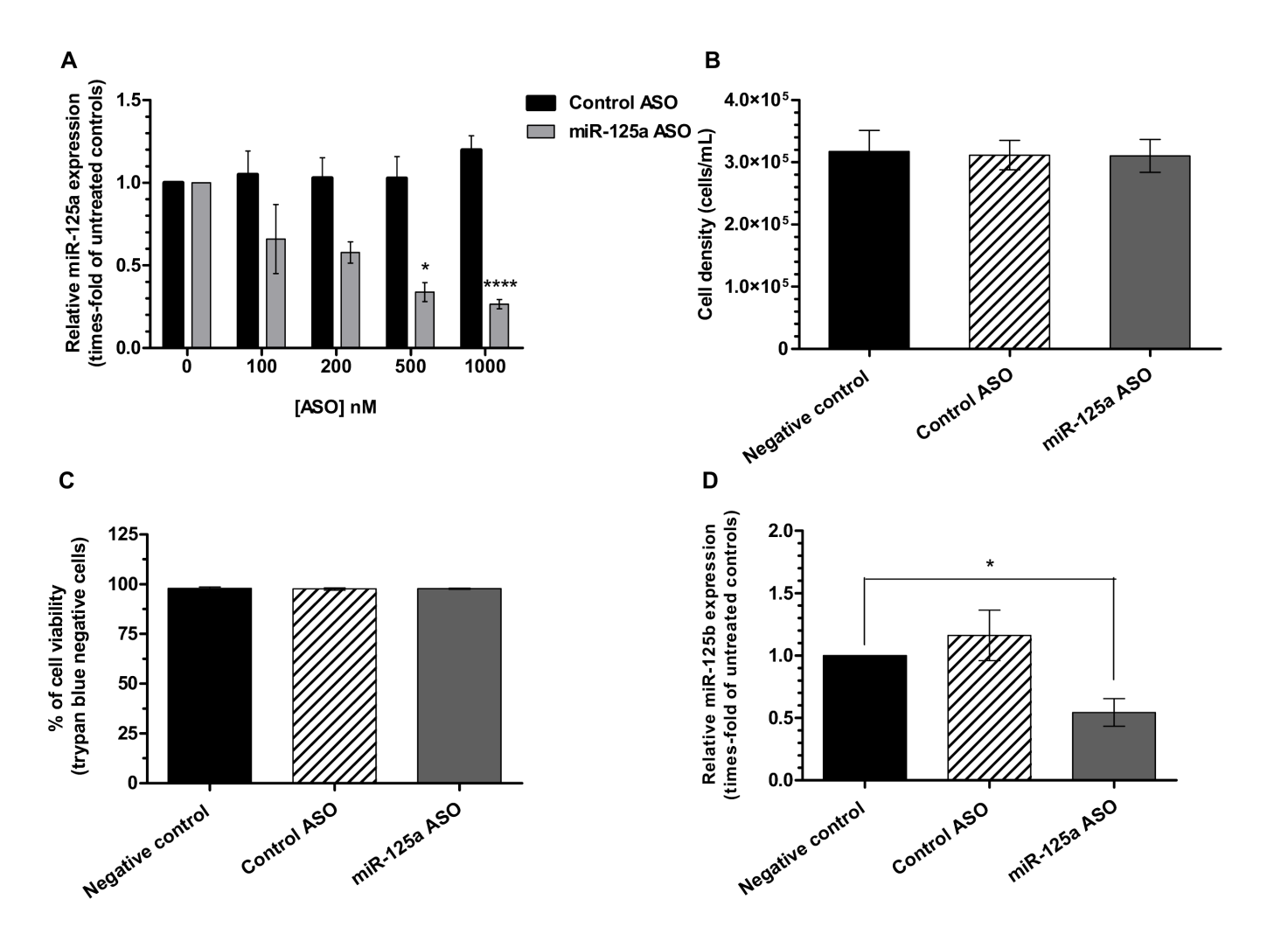

Supplement: Figure S4 — Efficiency of miR-125a inhibition in K562 cells. (A) Dose-response of miR-125a ASO. K562 cells were treated with increasing doses of ASO (100–1000 nM) for 48 hours and miR-125a expression was assessed by qPCR (n>2). (B–C) Effect of miR-125a inhibition on cell density and viability, respectively, of K562 cells after 48 hours of treatment with 1 µM ASO (n = 6). Negative controls are untreated cells. (D) Unspecific effect of miR-125a inhibition on miR-125b expression in K562 cells. (A–D) Data represent mean ± SEM. Statistical significance: *P<0.05; ***P<0.001; ****P<0.0001. (TIF) [file pone.0093404.s004.tif]

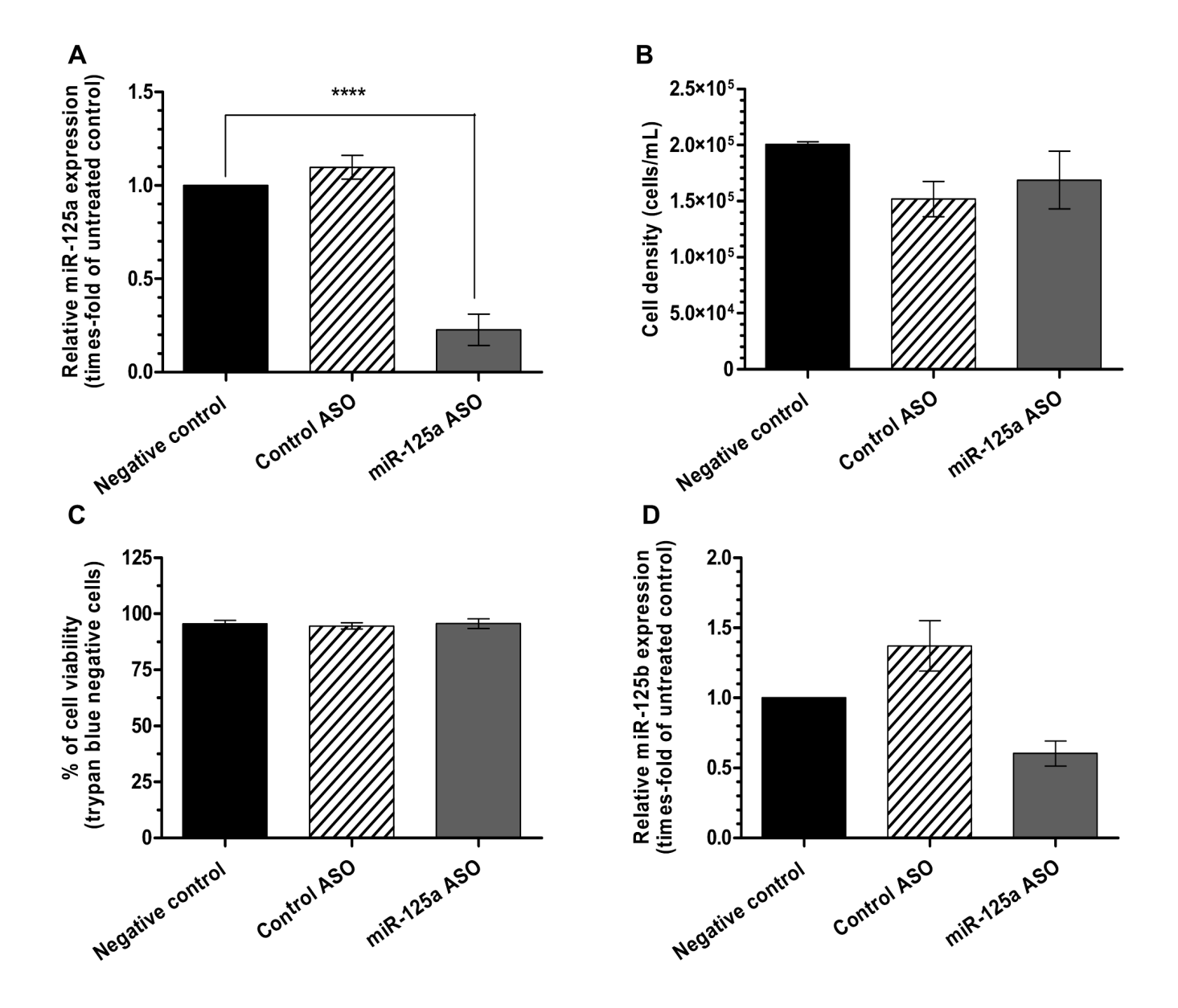

Supplement: Figure S5 — Efficiency of miR-125a inhibition in MDS-L cells. Cells were treated with 1 µM control and miR-125a ASO for 48 hours. (A) Changes in relative miR-125a expression, determined by qPCR. (B) Unspecific effect of miR-125a inhibition on miR-125b expression in MDS-L cells. (C–D) Effect of miR-125a inhibition on cell density and viability, respectively. (A–D) Data represent mean ± SEM of n = 3 experiments. Statistical significance: ****P<0.0001. (TIF) [file pone.0093404.s005.tif]

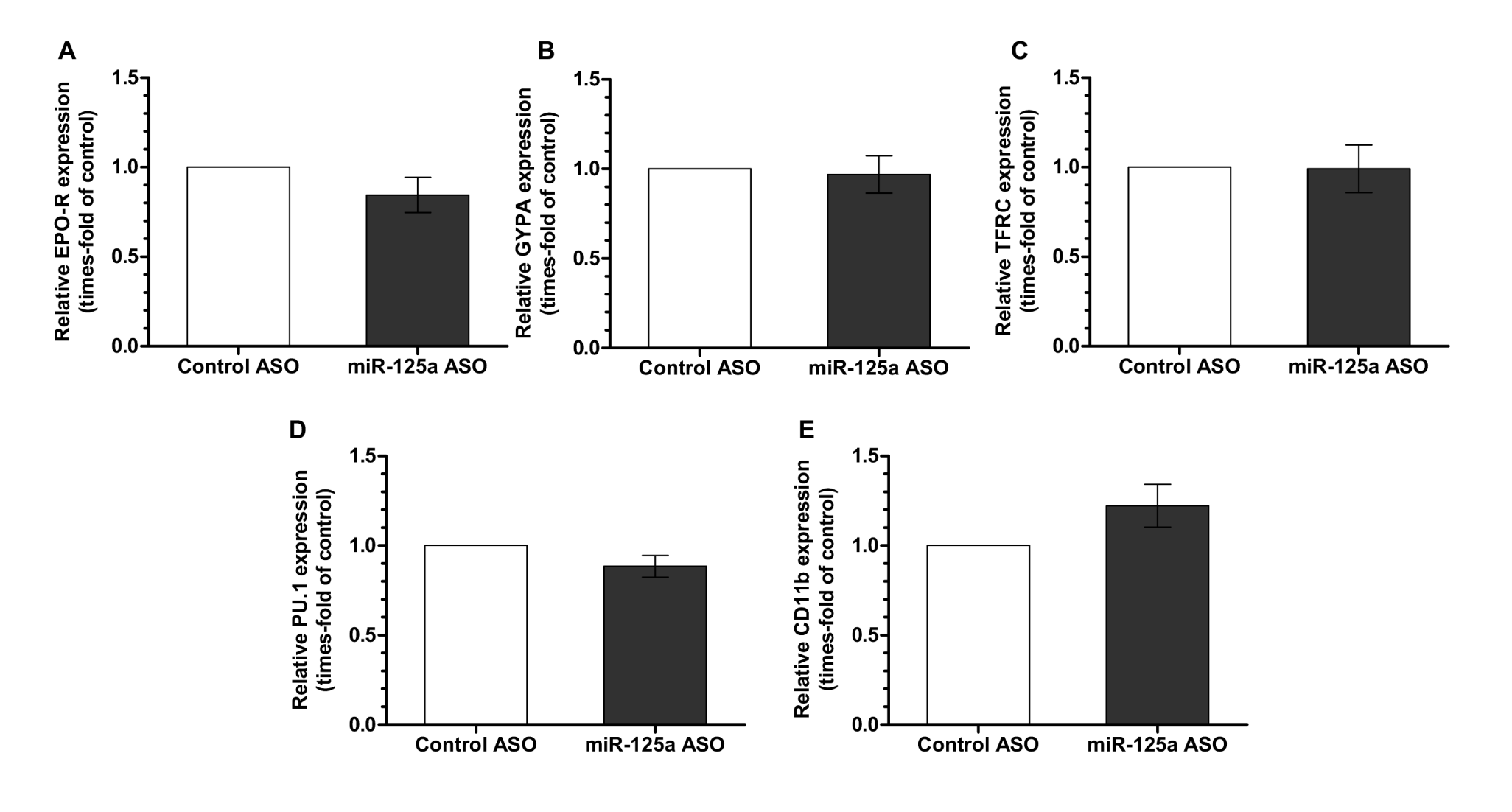

Supplement: Figure S6 — Effect of miR-125a inhibition on MDS-L cells differentiation. Relative expression levels of the differentiation markers (A) EPO-R, (B) GYPA, (C) CD71, (D) PU.1, (E) CD11b were determined by qPCR in 7-day colony samples previously treated for 48 hours with 1 µM ASOs. Data represent mean ± SEM of n = 8 independent experiments. (TIF) [file pone.0093404.s006.tif]

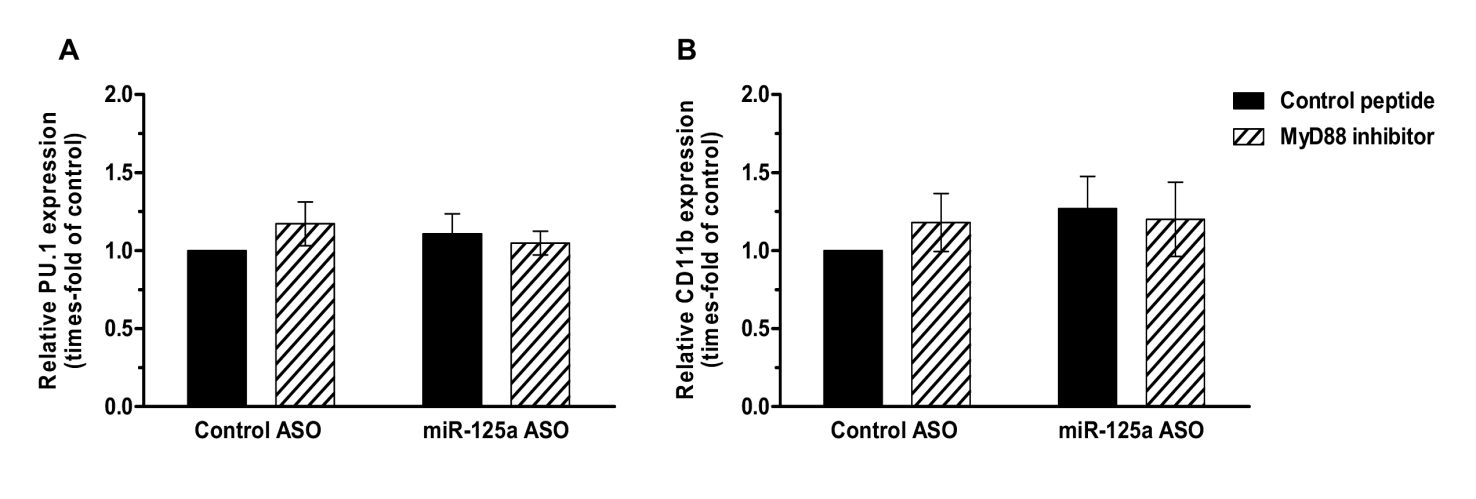

Supplement: Figure S7 — Effect of the inhibition of miR-125a and TLR2-NF-κB pathway in MDS-L cells. Relative expression levels of the myeloid differentiation markers (A) PU.1 and (B) CD11b were measured in colony samples by qPCR after a 7-day methylcellulose culture of cells previously treated with 1 µM ASO and 5 µM of the corresponding peptide. Black bars represent cells treated with control peptide, and striped bars represent cells treated with MyD88 inhibitor peptide. (TIF) [file pone.0093404.s007.tif]

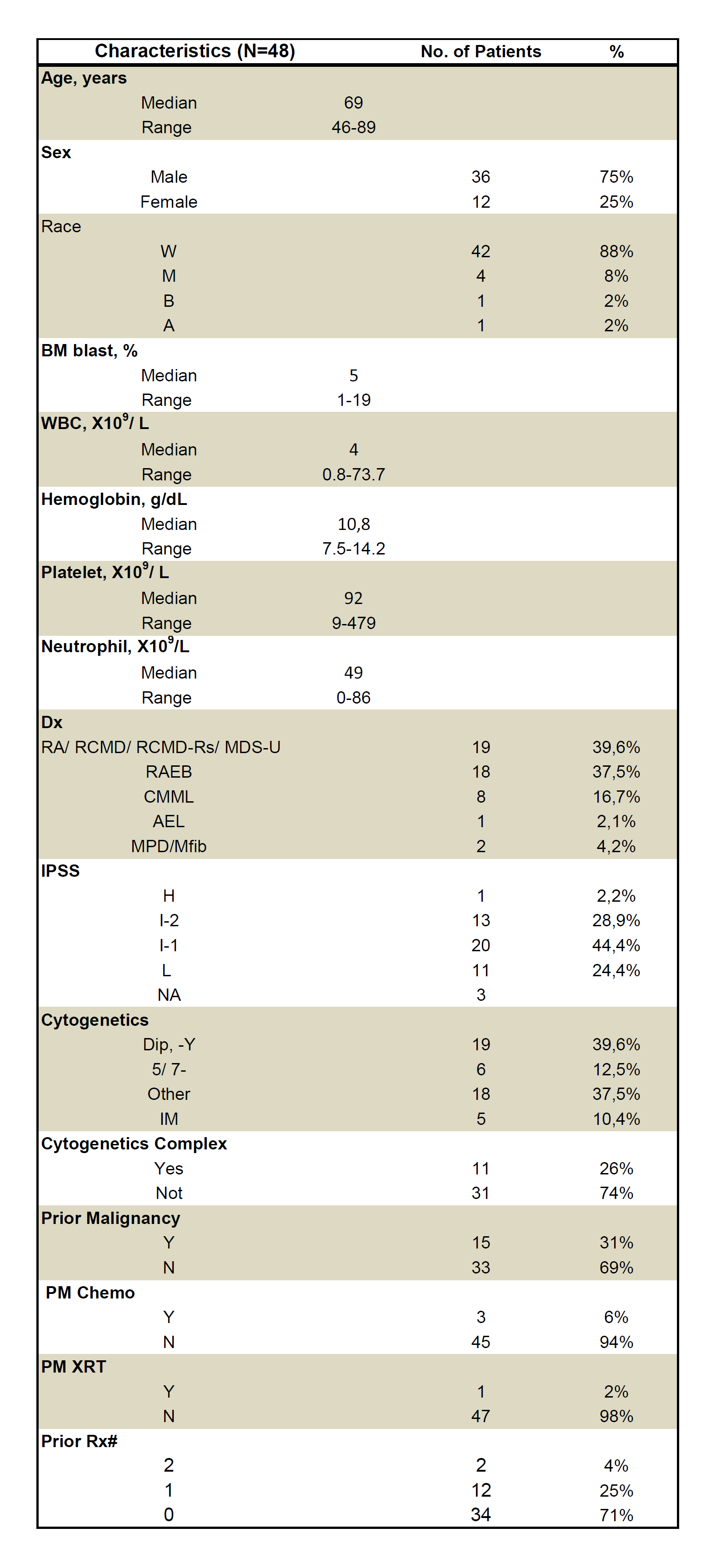

Supplement: Table S1 — Patient characteristics. (TIF) [file pone.0093404.s008.tif]

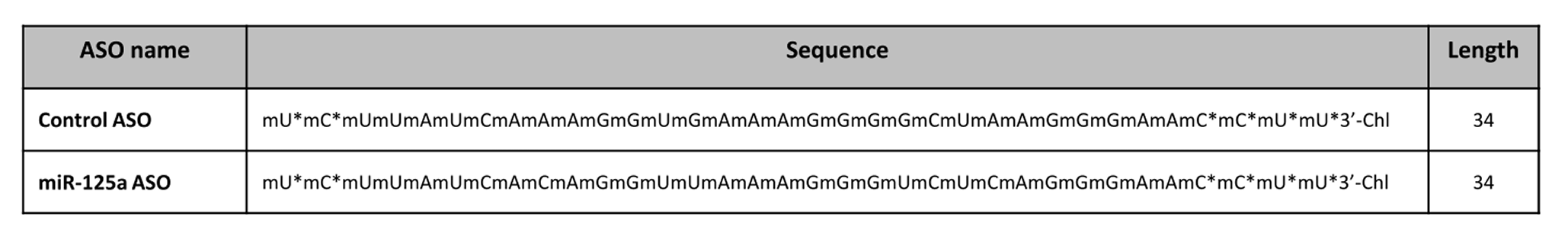

Supplement: Table S2 — Sequences of anti-sense oligonucleotides used for miR-125a inhibition assays. (m) = 2′O-methyl modification; (*) = phosphotiorate bond; (3′-Chl) = 3′ Cholesterol modification. (TIF) [file pone.0093404.s009.tif]
